# Autocrine GDF10 Inhibits Hepatic Stellate Cell Activation via BMPR2/ALK3 Receptor to Prevent Liver Fibrosis

**DOI:** 10.1002/advs.202500616

**Published:** 2025-03-24

**Authors:** Yinliang Zhang, Xiaochen Gai, Yuhui Li, Zuoyu Chen, Xi Zhang, Wei Qiao, Ping Qiu, Chunyuan Du, Sufang Sheng, Jingran Hao, Yujie Zhang, Heng Fan, Xiaorong Li, Ming Liu, Jun Zhang, Zhe Pan, Yongsheng Chang

**Affiliations:** ^1^ Key Laboratory of Immune Microenvironment and Disease (Ministry of Education) Tianjin Key Laboratory of Cellular Homeostasis and Disease Department of Physiology and Pathophysiology School of Basic Medical Sciences Tianjin Medical University Tianjin 300070 China; ^2^ School of Life Sciences Westlake University Hangzhou 310000 China; ^3^ Department of Minimally Invasive Esophageal Surgery Key Laboratory of Cancer Prevention and Therapy National Clinical Research Center for Cancer Tianjin Medical University Cancer Hospital and Institute Tianjin 300070 China; ^4^ Ningxia Key Laboratory of Stem Cell and Regenerative Medicine General Hospital of Ningxia Medical University Ningxia 750000 China; ^5^ Tianjin Key Laboratory of Retinal Functions and Diseases Tianjin Branch of National Clinical Research Center for Ocular Disease Eye Institute and School of Optometry Tianjin Medical University Eye Hospital Tianjin 300070 China; ^6^ Department of Endocrinology and Metabolism Tianjin Medical University General Hospital Tianjin 300070 China; ^7^ Department of Basic Medicine, School of Medicine Shihezi University Shihezi 832000 China; ^8^ Department of Endocrinology and Metabolism The Second Hospital of Shandong University Jinan 250033 China

**Keywords:** growth differentiation factor 10, hepatic stellate cells, liver fibrosis, transforming growth factor β

## Abstract

Hepatic stellate cells (HSCs) play a central role in the development of liver fibrosis, and their activation is controlled by a complex interplay of autocrine/paracrine signals within the liver microenvironment. Here, we show that growth differentiation factor 10 (GDF10) is specifically expressed by HSCs in both mouse and human livers, and its expression is reduced in activated HSCs. Loss of GDF10 function promotes HSC activation and exacerbates liver fibrosis in mice, while gain of GDF10 function alleviates this pathological condition. Mechanistically, autocrine GDF10 binds to BMPR2/ALK3 receptor to elicit SMAD1/5/8‐SMAD7 signaling pathway in HSCs. Activated SMAD1/5/8‐SMAD7 signaling pathway then inhibits the TGF‐β‐SMAD2/3 signaling transduction, which is essential for HSC activation. Moreover, recombinant GDF10 protein treatment suppresses HSC activation and alleviates liver fibrosis in mice. In conclusion, GDF10 is an autocrine suppressor of HSC activation and a potential therapeutic target for the treatment of liver fibrosis.

## Graphical abstract

Liver fibrosis is a growing global health burden with no specific treatment. HSC activation is the predominant driver of liver fibrogenesis and the current focus in antifibrotic drug discovery. This study identifies autocrine GDF10 activates BMPR2/ALK3‐SMAD1/5/8‐SMAD7 pathway to counteract the TGF‐β‐SMAD2/3 pathway in HSCs, thereby inhibiting HSC activation and preventing liver fibrosis.

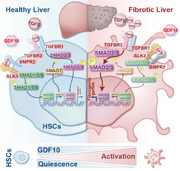

## 1 Introduction

Liver fibrosis is a reversible wound healing process characterized by excessive accumulation of extracellular matrix (ECM) proteins resulting from chronic liver injury caused by toxin exposure, viral hepatitis infection, metabolic dysfunction‐associated steatohepatitis (MASH), primary biliary cholangitis (PBC), and other liver diseases.^[^ ^1^, ^2^ ^]^ If left untreated, liver fibrosis can advance to cirrhosis and eventually lead to liver failure.^[^ ^3^ ^]^ Unfortunately, there are currently limited Food and Drug Administration‐approved drugs for the treatment of liver fibrosis. Therefore, it is essential to investigate the mechanisms underlying liver fibrosis and identify potential therapeutic targets.

HSCs, located in the perisinusoidal space of the liver between hepatocytes (HCs) and liver sinusoidal endothelial cells (LSECs), are the primary source of ECM and play a vital role in liver fibrosis.^[^ ^4^ ^]^ Following liver injury, HSCs become activated, transdifferentiating from quiescent cells to myofibroblasts, which are proliferative and characterized by enhanced ECM production.^[^ ^4^ ^]^ Hepatic autocrine/paracrine signaling is essential for tissue homeostasis, and its dysfunction contributes to liver disease progression.^[^ ^5^ ^]^ Recently, several autocrine/paracrine factors derived from injured HCs, activated HSCs, and immune cells have been identified to induce HSC activation during liver fibrosis.^[^ ^2^, ^6^ ^]^ However, the intrahepatic signals that modulate HSC activation remain largely elusive.

GDF10, also known as BMP3b, belongs to the transforming growth factor beta (TGF‐β) superfamily.^[^ ^7^ ^]^ It was initially considered a redundant growth factor‐like molecule due to the absence of apparent developmental abnormalities in the *Gdf10* knockout mice.^[^ ^7^ ^]^ However, recent studies have shown that GDF10 is involved in many pathological conditions, such as stroke,^[^ ^8^ ^]^ obesity,^[^ ^9^ ^]^ age‐related sarcopenia,^[^ ^10^ ^]^ and fibrotic lung disease.^[^ ^11^ ^]^ Moreover, a recent study has suggested the involvement of GDF10 in hepatocyte lipid accumulation.^[^ ^12^ ^]^ However, the distinct role of GDF10 in the liver and its expression profiles remain to be understood. Notably, GDF10 is specifically expressed by mesenchymal progenitor cells in adipose tissue and skeletal muscle.^[^ ^10^, ^13^ ^]^ As stem‐like cells, HSCs have been described as liver‐resident mesenchymal progenitor cells,^[^ ^14^ ^]^ implying the potential biological significance of GDF10 in HSCs.

By analyzing transcriptome datasets from mouse fibrotic livers, we identified GDF10 as a liver fibrosis‐associated cytokine. We found that GDF10 is specifically expressed by HSCs in the liver. During HSC activation, GDF10 expression was decreased. Moreover, the loss of GDF10 function promoted TGF‐β‐induced HSC activation, while the gain of GDF10 function prevented TGF‐β‐induced HSC activation. We subsequently dissected the significance of GDF10 in liver fibrogenesis and explored its potential clinical significance in the treatment of liver fibrosis in mice.

## 2 Results

### 2.1 Hepatic GDF10 Is Specifically Expressed by HSCs and Is Downregulated in Activated HSCs

To gain insight into the fibrotic process in the liver, we first analyzed transcriptome datasets from mouse fibrotic livers induced by carbon tetrachloride (CCl4) (GSE130123),^[^ ^15^ ^]^ PBC (GSE179993),^[^ ^16^ ^]^ methionine‐choline deficient (MCD) diet (GSE156918),^[^ ^17^ ^]^ and high‐fat, high‐fructose, high‐cholesterol (AMLN) diet (GSE119340).^[^ ^18^ ^]^ We identified 171 common differentially expressed genes (DEGs) between normal and fibrotic livers in all these datasets (Figure S1A, Supporting Information). Using biological process enrichment analysis, we did find that these genes were linked to liver fibrosis‐related biological processes, such as collagen fibril organization, extracellular matrix organization, and wound healing (Figure S1B, Supporting Information). Further cellular component enrichment analysis revealed that these DEGs were enriched in extracellular space, suggesting intrahepatic crosstalk alteration in the fibrotic liver (Figure S1C, Supporting Information). Among the extracellular region‐related DEGs, GDF10 was identified as a novel liver fibrosis‐associated cytokine (Figure S1C, Supporting Information).

**Figure 1:**
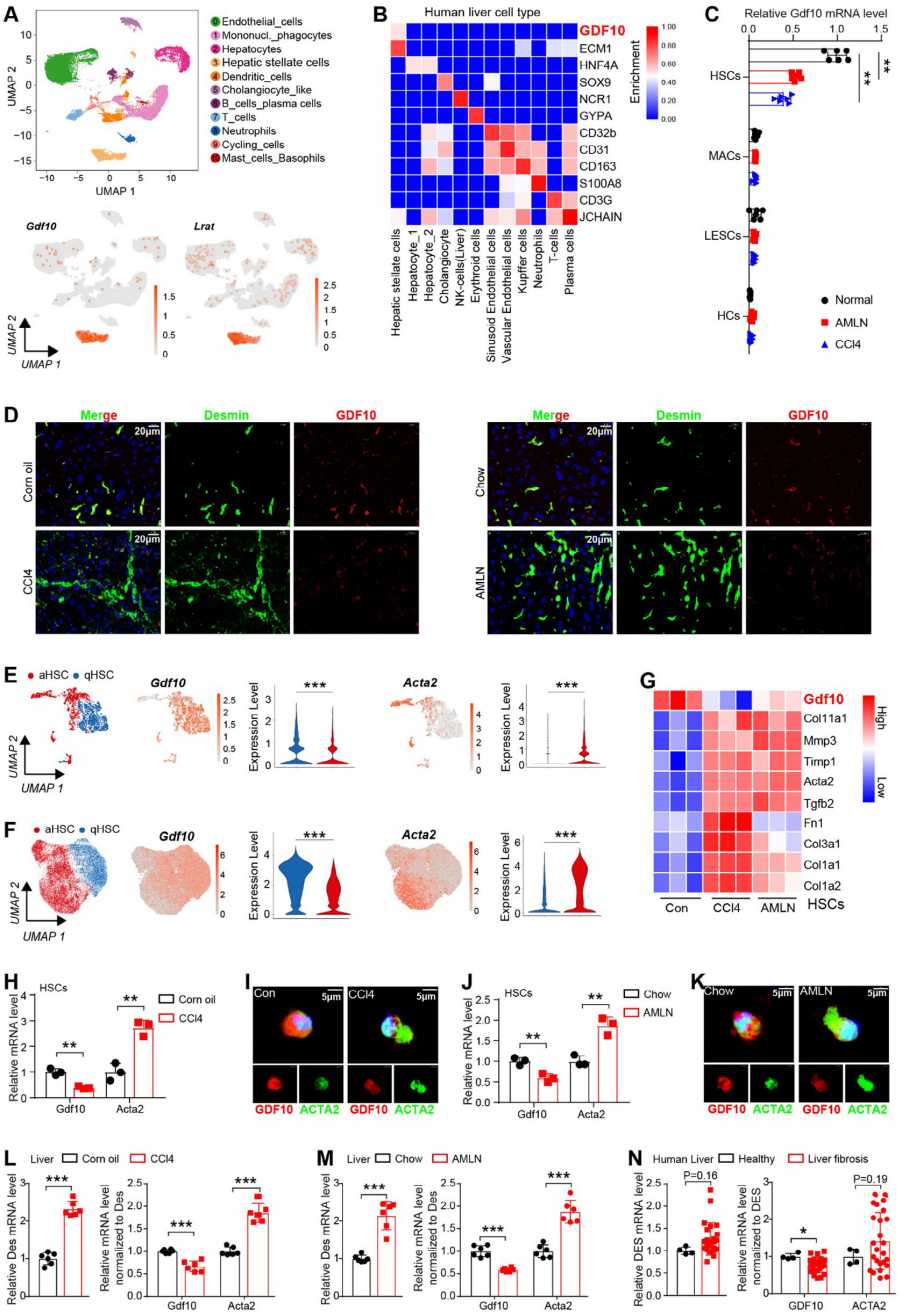
Hepatic GDF10 is specifically expressed by HSCs and is downregulated in activated HSCs. A) UMAP visualization of the cell type expresses *Gdf10* in the liver (GSE218299). B) Cell type enrichment of GDF10 in human liver (Human Protein Atlas database). C) qPCR analysis of *Gdf10* mRNA levels in LSECs, MACs, HSCs, and HCs from the normal, CCl4‐induced fibrotic, and AMLN diet‐induced fibrotic liver. D) IF staining analysis of GDF10 and Desmin protein levels in the liver, scale bars, 20 µm. E,F) UMAP visualization of the *Gdf10* and *Acta2* mRNA levels in the qHSCs and aHSCs, data from GSE218299 (E) and GSE171904 (F). G) Heat map representation of the indicated genes in HSCs from CCl4 treated or AMLN diet‐fed mice (GSE134512). H,I) qPCR (H) (*n* = 6) and IF staining (I) analysis of indicated genes in HSCs isolated from normal and CCl4‐induced fibrotic liver, scale bars, 5 µm. J,K) qPCR (J) (*n* = 6) and IF staining (K) analysis of indicated genes in HSCs isolated from normal and AMLN diet‐induced fibrotic liver, scale bars, 5 µm. L,M) qPCR analysis of *Desmin* (*Des*) mRNA levels (normalized to 36B4) and *Gdf10* and *Acta2* mRNA levels (normalized to *Des*) in fibrotic liver. N) Analysis of *DES* mRNA levels and *GDF10* and *ACTA2* mRNA levels (normalized to *DES*) in human health and fibrotic liver tissues from dataset GSE159676. Data are mean ± SEM. *p < 0.05, **p < 0.01, ***p < 0.001 by the two‐tailed Student's *t*‐test.

By analyzing the single‐cell RNA‐seq data (GSE218299)^[^ ^19^ ^]^ and the Human Protein Atlas database, we found that HSCs are the cell origin expressing *GDF10* in both mouse and human livers (**Figure** **1**A,B; Figure S2A,B, Supporting Information). Subsequently, we isolated HSCs, HCs, LSECs, and macrophages (MACs) from normal and fibrotic mouse livers and confirmed that *Gdf10* was predominantly expressed in HSCs (Figure 1C; Figure S2C, Supporting Information). Moreover, GDF10 colocalized with Desmin, a marker gene expressed in HSCs regardless of their activation status, in normal and fibrotic mouse livers (Figure 1D). These data indicate that hepatic GDF10 is specifically expressed by HSCs.

To uncover *Gdf10* expression profile in HSCs in‐depth, we extracted all HSC transcriptomic information from single‐cell RNA‐seq data of fibrotic mouse livers induced by bile duct ligation and CCl4 (GSE171904) and AMLN diet (GSE218299).^[^ ^19^, ^20^ ^]^ HSCs were divided into two subpopulations: quiescent HSCs (qHSCs) and activated HSCs (aHSCs) (Figure 1E,F; Figure S2D,E, Supporting Information). To our surprise, *Gdf10* expression was downregulated in aHSCs compared to qHSCs (Figure 1E,F). Consistently, RNA‐seq transcriptomic profiling of HSCs from normal and fibrotic mouse livers showed that *Gdf10* expression was downregulated in HSCs of fibrotic mouse livers^[^ ^21^ ^]^ (Figure 1G). Furthermore, our qPCR and IF staining analysis validated the decreased expression of GDF10 in HSCs from fibrotic mouse livers (Figure 1H–K). Liver fibrosis involves the proliferation of activated HSCs.^[^ ^2^ ^]^ By examining the Desmin and ACTA2 (a marker of activated HSCs) positive cells in normal and fibrotic mouse livers, we confirmed that liver fibrosis is associated with an elevated quantity of activated HSCs (Figure S2F,G, Supporting Information). Although *Gdf10* mRNA was increased in fibrotic livers, the number of HSCs was increased, leading to a decrease in the mRNA of *Gdf10* per HSC in fibrotic livers (Figure 1L,M). Consistently, this result was confirmed through the analysis of GSE159676 dataset for human fibrotic livers (Figure 1N).^[^ ^22^ ^]^ Together, our data suggest that GDF10 is specifically expressed in HSCs and is downregulated in activated HSCs.

### 2.2 GDF10 Inhibits TGF‐β‐Induced HSC Activation

**Figure 2:**
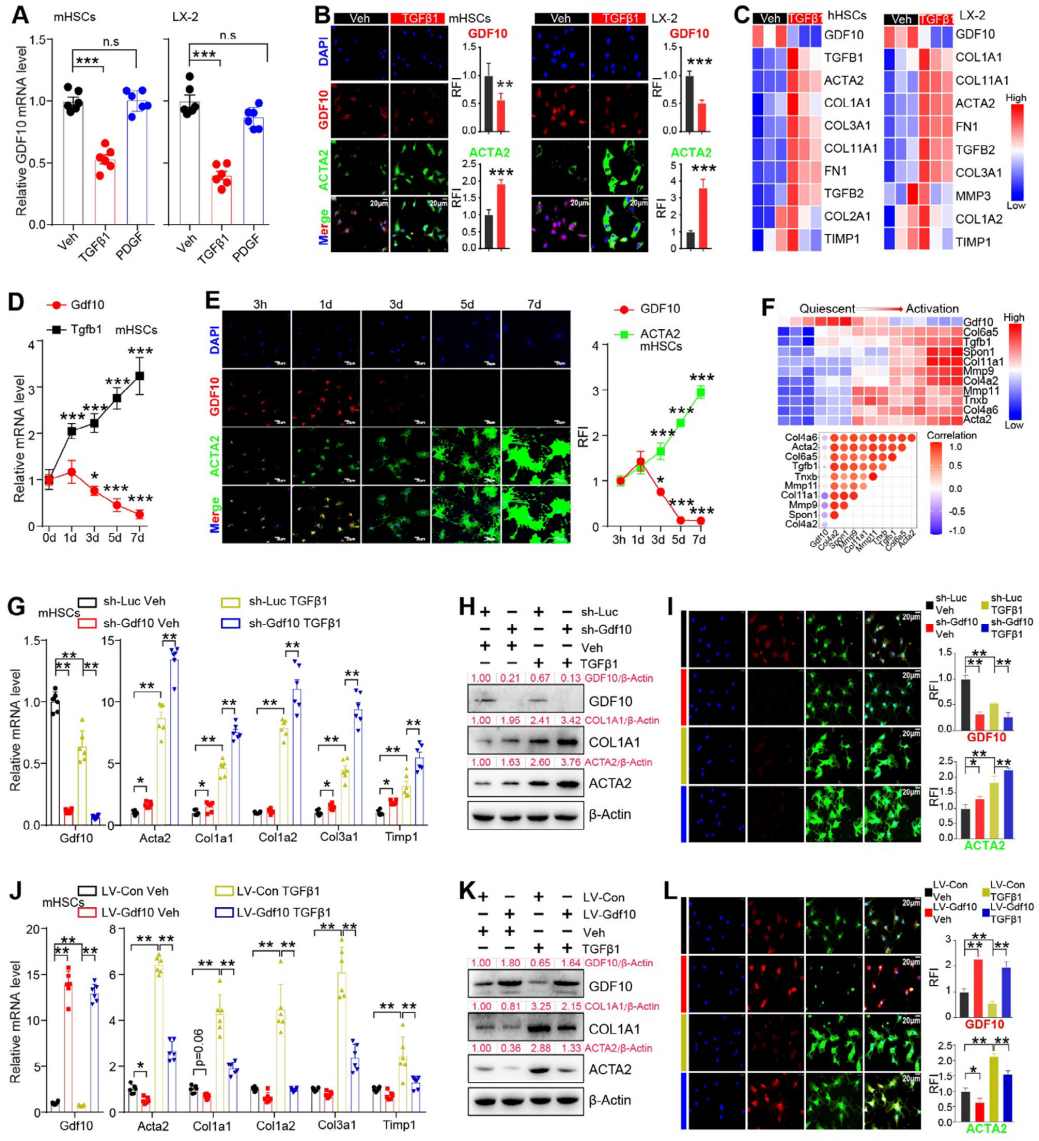
GDF10 inhibits TGF‐β‐induced HSC activation. A) qPCR analysis of *GDF10* mRNA levels in primary mouse HSCs and LX‐2 cells treated with TGF‐β1 (2 ng mL^−1^), PDGF (20 ng mL^−1^), or vehicle (Veh) for 24 h (*n* = 6). B) IF staining analysis of GDF10 protein levels in primary mouse HSCs and LX‐2 cells treated with TGF‐β1 (2 ng mL^−1^) or vehicle for 24 h (*n* = 6), the relative fluorescence intensity (RFI) of ACTA2 and GDF10 is also shown, scale bars, 20 µm. C) Heat map representation of the indicated genes in primary human HSCs (GSE119606) and LX‐2 cells (GSE151251) treated with TGF‐β1 or vehicle. D, E) qPCR analysis of *Gdf10* and *Tgfb1* mRNA levels (D) (*n* = 6) and IF staining analysis of GDF10 and ACTA2 protein levels (E) (*n* = 3) in HSCs during the culture activation. F) Heat map representation of the indicated genes in HSCs during the culture activation (GSE116987). G–I) qPCR (G) (*n* = 6), Western blot (H), and IF staining (I) (*n* = 3) analysis of indicated genes in primary mouse HSCs infected with LV‐sh‐Luc or LV‐sh‐Gdf10 for 24 h and then treated with TGF‐β1 (2 ng mL^−1^) or vehicle for another 24 h, scale bars, 20 µm. J–L) qPCR (J) (*n* = 6), Western blot (K), and IF staining (L) (*n* = 3) analysis of indicated genes in primary mouse HSCs infected with LV‐Con or LV‐Gdf10 for 24 h and then treated with TGF‐β1 (2 ng mL^−1^) or vehicle for another 24 h, scale bars, 20 µm. Data are mean ± SEM. *p < 0.05, **p < 0.01, ***p < 0.001 by the two‐tailed Student's *t*‐test (B,D,E), one‐way ANOVA (A, G (left), J (left), I, and L), or two‐way ANOVA (G (right) and J (right)).

Next, we investigated the significance of GDF10 in HSC activation. TGF‐β and platelet‐derived growth factor (PDGF) are multifunctional cytokines that play a critical role in HSC activation and liver fibrosis. TGF‐β primarily facilitates the transdifferentiation of HSCs into myofibroblast‐like cells, whereas PDGF primarily promotes HSC proliferation.^[^ ^23^ ^]^ We subjected primary mouse HSCs and human HSCs (LX‐2 cells) to TGF‐β1 or PDGF proteins. PDGF protein treatment did not affect *GDF10* expression (**Figure** **2**A), while TGF‐β1 treatment suppressed *GDF10* expression (Figure 2A,B). By analyzing the GSE119606 and GSE151251 datasets,^[^ ^24^ ^]^ we observed reduced *GDF10* mRNA in TGF‐β1‐treated primary human HSCs and LX‐2 cells (Figure 2C). Culture activation is another widely used approach to differentiate primary HSCs into myofibroblast‐like cells.^[^ ^25^ ^]^ During the culture activation of primary mouse HSCs, there was a gradual upregulation in TGF‐β1 expression and a concurrent decrease in GDF10 expression (Figure 2D,E). Consistently, we observed the reduction of *GDF10* mRNA in culture‐activated primary mouse HSCs by analyzing the GSE116987 dataset (Figure 2F).^[^ ^26^ ^]^ Hence, we postulated that GDF10 might be involved in the activation of HSCs induced by TGF‐β.

To assess the impact of GDF10 on TGF‐β‐induced HSC activation, *Gdf10* targeted shRNA lentivirus (LV‐sh‐Gdf10) was utilized to deplete GDF10 (Figure 2G–I). GDF10 depletion promoted TGF‐β1‐stimulated expression of fibrogenic genes, such as *Acta2*, collagen type I alpha 1 chain (*Col1a1*), *Col1a2*, *Col3a1*, and TIMP metallopeptidase inhibitor 1 (*Timp1*) in primary mouse HSCs, which also serve as marker genes for HSC activation (Figure 2G). In addition, GDF10‐depleted HSCs showed enhanced ACTA2 and COL1A1 protein levels and myofibroblast‐like phenotype induced by TGF‐β1 (Figure 2H,I). Consistently, lentivirus‐mediated overexpression of GDF10 intercepted TGF‐β1‐stimulated HSC activation, including primary mouse HSCs and JS1 cells (a mouse HSC cell line) (Figure 2J–L; Figure S3A–C, Supporting Information). Additionally, recombinant GDF10‐Fc protein (containing the Fc fragment and the mature region of GDF10) treatment inhibited TGF‐β1‐stimulated HSC activation without altering PDGF‐induced HSC proliferation (Figure S3D–M, Supporting Information). These data suggest that GDF10 plays an inhibitory role in TGF‐β‐induced HSC activation.

### 2.3 *Gdf10* Deletion Promotes HSC Activation and Accelerates Liver Fibrosis Progression in Mice

**Figure 3:**
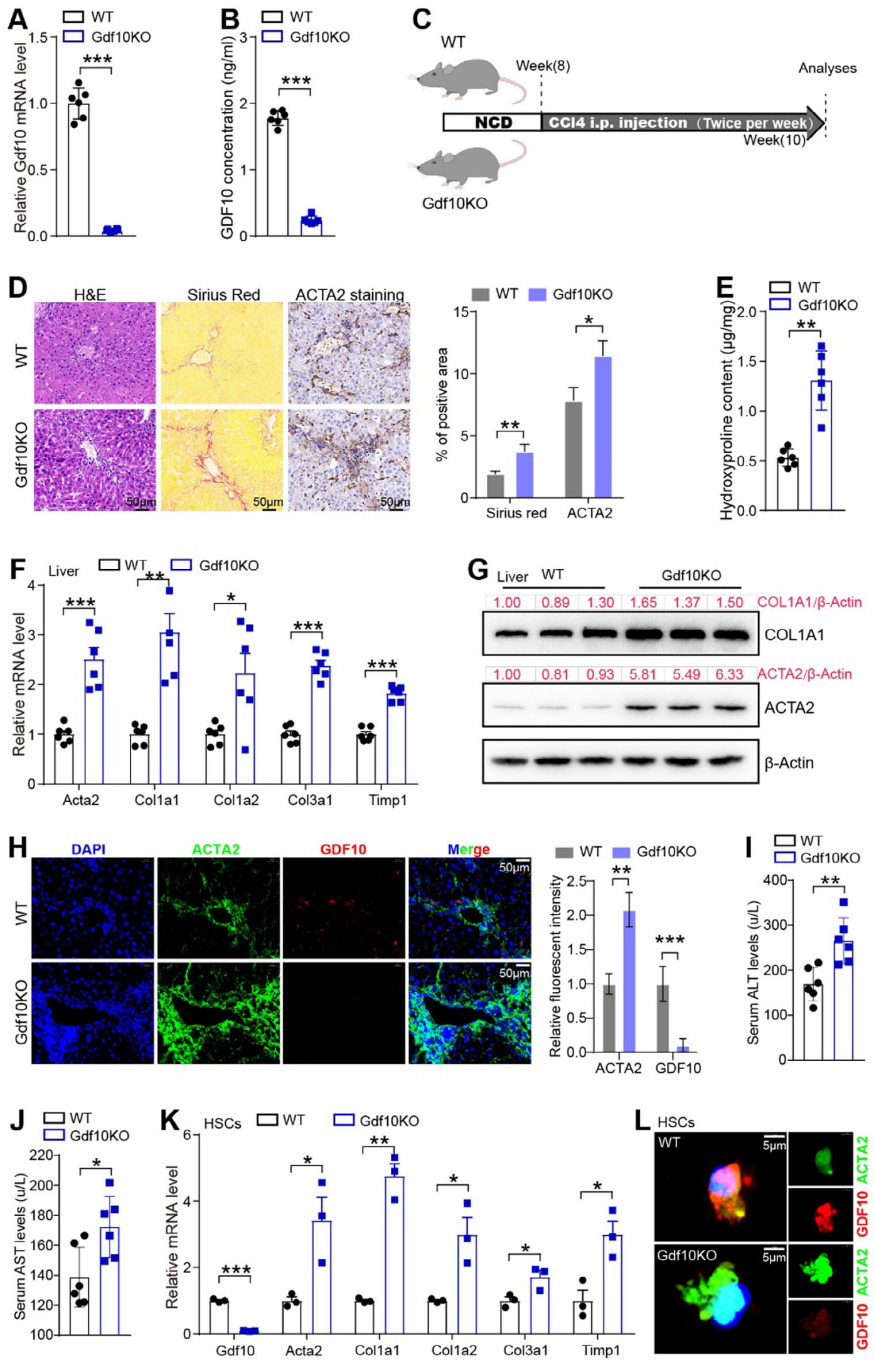
*Gdf10* knockout promotes HSC activation and exacerbates CCl4‐induced liver fibrosis. A) qPCR analysis of *Gdf10* mRNA levels in the HSCs isolated from 12‐week‐old male WT and Gdf10KO mice. B) ELISA analysis of serum GDF10 content in WT and Gdf10KO mice (*n* = 6). C) Schematic drawing of the experimental procedure. D) Representative images of H&E, Sirius Red, and ACTA2 IHC staining, scale bars, 50 µm (*n* = 3). E) Hydroxyproline assay analysis of total liver collagen content (*n* = 6). F‐H) qPCR (F) (*n* = 6), Western blot (G), and IF staining (H) (*n* = 3) analysis of indicated genes in the liver, scale bars, 50 µm. I, J) Measurement of serum ALT (I) and AST (J) levels of WT and Gdf10KO mice. K, L) qPCR (K) (*n* = 3) and IF staining (L) analysis of indicated genes in the primary mouse HSCs, scale bars, 5 µm. Data are mean ± SEM. *p < 0.05, **p < 0.01, ***p < 0.001 by the two‐tailed Student's *t*‐test.

The inhibitory effects of GDF10 on TGF‐β‐induced HSC activation impelled us to examine the role of GDF10 in liver fibrosis. To this end, we generated global *Gdf10* knockout (Gdf10KO) mice (**Figure** **3**A,B; Figure S4A,B, Supporting Information).

Next, Gdf10KO and their littermate wild‐type (WT) mice were given short‐term injections of CCl4 (2 weeks) (Figure 3C) or subjected to bile duct ligation (BDL) (2 weeks) to induce liver fibrosis. Sirius Red staining showed that Gdf10KO mice had exacerbated liver fibrosis compared to WT mice (Figure 3D; Figure S4C, Supporting Information). Consistently, Gdf10KO mice had higher hepatic hydroxyproline levels than WT mice (Figure 3E; Figure S4D, Supporting Information). The expression levels of fibrogenic genes in the livers of Gdf10KO mice were also increased (Figure 3F–H; Figure S4E, Supporting Information). Furthermore, aggravated liver injury in Gdf10KO mice, evidenced by elevated serum aspartate aminotransferase (AST) and alanine aminotransferase (ALT), further confirmed the exacerbation of liver fibrosis (Figure 3I,J; Figure S4F, Supporting Information).

MASH is emerging as a leading cause of progressive liver fibrosis and end‐stage liver disease.^[^ ^27^ ^]^ Therefore, we fed Gdf10KO and WT mice an AMLN diet for 28 weeks or an MCD diet for 4 weeks to induce MASH‐related fibrosis. Compared to WT littermates, Gdf10KO mice exhibited heightened Sirius Red staining in the liver (Figure S4G,K, Supporting Information), increased hepatic hydroxyproline content (Figure S4H,L, Supporting Information), upregulated fibrotic gene expression in the liver (Figure S4I,M, Supporting Information), and elevated serum ALT and AST levels (Figure S4J,N, Supporting Information), consistent with the evidence from CCl4‐ and BDL‐induced fibrosis models.

To investigate whether the profibrotic effect of *Gdf10* knockout was associated with HSC activation, we isolated HSCs from the livers of CCl4‐treated WT and Gdf10KO mice. As a result, HSC activation was enhanced in Gdf10KO mice (Figure 3K,L). In addition, we also found a slight increase in HSC activation in Gdf10KO mice under normal conditions at 2 months of age. However, Gdf10KO mice did not progress to liver fibrosis (Figure S5A–D, Supporting Information). By 12 months of age, Gdf10KO mice developed spontaneous liver fibrosis with activated HSCs (Figure S5E–K, Supporting Information). Therefore, these data suggest that *Gdf10* deficiency promotes HSC activation and accelerates liver fibrosis.

### 2.4 *Gdf10* Transgene Inhibits HSC Activation and Attenuates Liver Fibrosis in Mice

**Figure 4:**
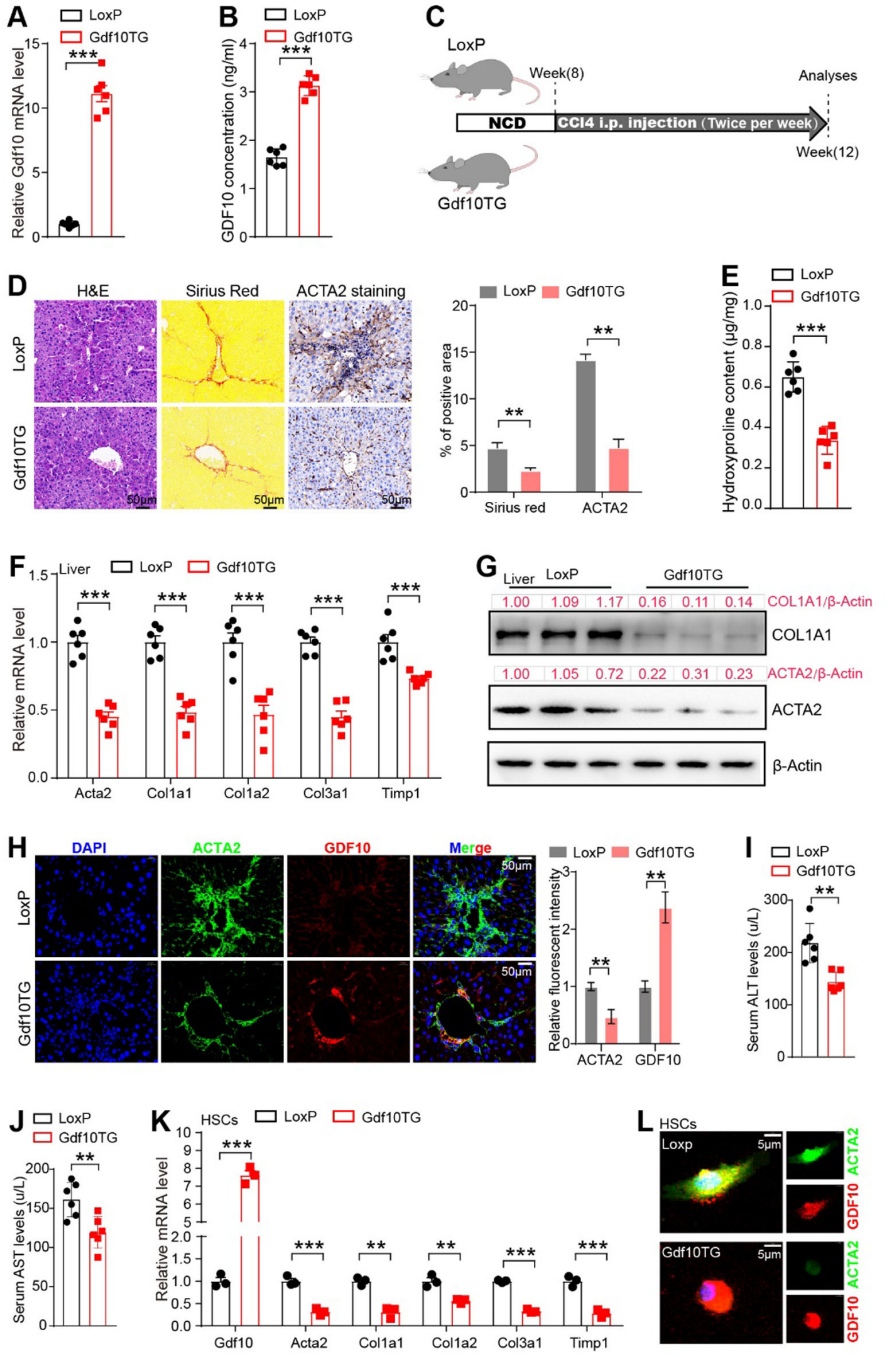
HSC‐specific *Gdf10* transgene inhibits HSC activation and attenuates liver fibrosis in mice. A) qPCR analysis of *Gdf10* mRNA levels in the HSCs isolated of 12‐week‐old male *Rosa26‐Gdf10* ^flox/flox^ (LoxP) and *Rosa26‐Gdf10* ^flox/flox^; Lrat‐Cre (Gdf10TG) mice (*n* = 6). B) ELISA analysis of serum GDF10 content in LoxP and Gdf10TG mice (*n* = 6). C) Schematic drawing of the experimental procedure. D) Representative images of H&E, Sirius Red, and ACTA2 IHC staining, scale bars, 50 µm (*n* = 3). E) Hydroxyproline assay analysis of total liver collagen content (*n* = 6). F–H) qPCR (F) (*n* = 6), Western blot (G), and IF staining (H) (*n* = 3) analysis of indicated genes in the liver, scale bars, 50 µm. I,J) Measurement of serum ALT (I) and AST (J) levels of LoxP and Gdf10TG mice. K,L) qPCR (K) (*n* = 3) and IF staining (L) analysis of indicated genes in the primary mouse HSCs, scale bars, 5 µm. Data are mean ± SEM. *p < 0.05, **p < 0.01, ***p < 0.001 by the two‐tailed Student's *t*‐test.

To further explore the role of GDF10 in HSC activation and liver fibrosis, we generated HSC‐specific *Gdf10* transgenic (Gdf10TG) mice by crossing Lrat‐Cre mice with the *Gdf10* LoxP mice harboring the LoxP‐Stop‐LoxP‐Gdf10 cassette in the *Rosa26* locus (**Figure** **4**A,B; Figure S6A,B, Supporting Information). We then induced liver fibrosis in Gdf10TG and their littermate LoxP mice, including CCl4‐, BDL‐, AMLN diet‐, and MCD diet‐induced fibrosis models. In these fibrotic mouse models, we consistently found that *Gdf10* transgene reduced Sirius red staining in the liver (Figure 4C,D; Figure S6C,G,K, Supporting Information), reduced hepatic hydroxyproline content (Figure 4E; Figure S6D,H,L, Supporting Information), and inhibited fibrotic gene expression in the liver (Figure 4F–H; Figure S6E,I,M, Supporting Information). In addition, *Gdf10* transgene effectively attenuated liver injury, as demonstrated by a decrease in serum ALT and AST levels (Figure 4I,J; Figure S6F,J,N, Supporting Information). Moreover, HSCs isolated from Gdf10TG mice showed decreased expression of HSC activation marker genes (Figure 4K,L). These results demonstrate that GDF10 mitigates liver fibrosis and inhibits HSC activation in mice.

### 2.5 BMPR2/ALK3 Is a Receptor for GDF10 in HSCs

**Figure 5:**
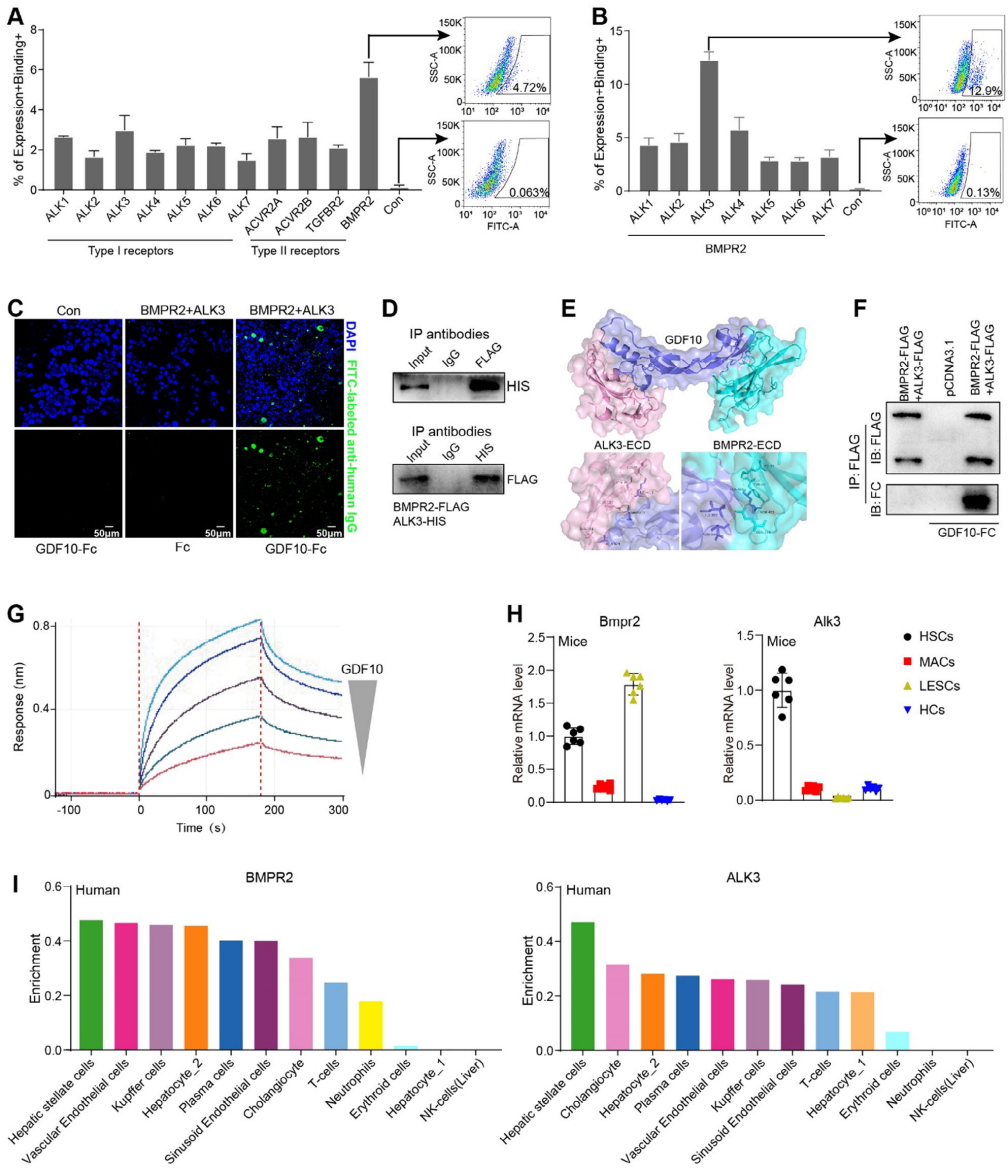
Characterization of the interaction between GDF10 and BMPR2/ALK3. A) Percentage of expression^+^ binding^+^ transfectants of HEK293 cells expressing a single TGF‐β superfamily receptor incubated with GDF10‐Fc. B) Percentage of expression^+^ binding^+^ transfectants of HEK293 cells expressing BMPR2 and type I TGF‐β receptor incubated with GDF10‐Fc. C) HEK293 cells were transfected to express BMPR2 and ALK3 and then incubated with GDF10‐Fc, scale bars, 50 µm. D) Association of BMPR2 with ALK3. HEK293 cells were transfected to express BMPR2‐FLAG and ALK3‐HIS. E) The overall structure of mature GDF10 with the extracellular domains (ECD) of mouse BMPR2 and ALK3 (GDF10: blue‐purple, BMPR2: pink‐green, ALK3: pink). F) Western blot analysis of cell lysates from HEK293 cells transfected to express C‐terminal FLAG‐tagged BMPR2 and FLAG‐tagged ALK3 incubated with GDF10‐Fc and then immunoprecipitated with anti‐FLAG antibody. G) Biacore sensorgrams and binding kinetics were determined by SPR spectroscopy for GDF10 with the ECD of mouse BMPR2 and ALK3. H) qPCR analysis of the expression *Bmpr2* and *Alk3* in mouse LSECs, MACs, HSCs, and HCs (*n* = 6). I) Cell type enrichment of BMPR2 and ALK3 in human liver (Human Protein Atlas database).

TGF‐β superfamily members signal through interactions with type I and type II heterodimer receptors, leading to phosphorylation of receptor‐regulated (R‐) SMADs (SMAD1, SMAD2, SMAD3, SMAD5, and SMAD8) or activation of SMAD‐independent pathways.^[^ ^28^ ^]^ GDF10 has been reported to signal via the SMAD‐dependent pathways.^[^ ^10^, ^29^, ^30^ ^]^ Accordingly, we hypothesized that GDF10 binds to specific TGF‐β superfamily receptors to inhibit HSC activation. To test this hypothesis, we transfected the equivalent cDNA of each member of the TGF‐β superfamily receptors into HEK293 cells to investigate their interaction with GDF10. As a result, GDF10 bound to BMPR2 with higher affinity than other receptors (**Figure** **5**A). BMPR2 is a type II TGF‐β superfamily receptor. Some ligands of the TGF‐β superfamily have higher binding affinity when both type I and type II receptors are present.^[^ ^31^ ^]^ Thus, we co‐transfected *Bmpr2* cDNA with different type I receptor cDNAs into HEK293 cells. GDF10 exhibited higher binding efficiency to cells with overexpressed *Bmpr2* and *Alk3* compared to other receptor types overexpressed cells (Figure 5B). The potential binding of GDF10‐Fc to cells overexpressing *Bmpr2* and *Alk3* was validated by the IF staining (Figure 5C). Furthermore, BMPR2‐FLAG and ALK3‐His co‐immunoprecipitated with each other (Figure 5D). Importantly, we found that the expression levels of both *Bmpr2* and *Alk3* do not decrease during HSC activation, suggesting that these receptors are persistently available for ligand binding (Figure S7A, Supporting Information). Therefore, GDF10 likely binds to BMPR2/ALK3 heterodimeric receptor to inhibit HSC activation.

To further evaluate the effectiveness of GDF10 binding to BMPR2/ALK3, we performed the molecular dynamics simulation analysis. Using homology modeling structures of GDF10 mature domain (residues 372–478), BMPR2 extracellular domain (residues 27–140), and ALK3 extracellular domain (residues 51–152) from Alphafold2, we simulated the interaction between GDF10 and BMPR2/ALK3 heterodimer (Figure 5E). This interaction was further confirmed via immunoprecipitation analysis (Figure 5F). Subsequently, we engineered a heterodimeric fusion protein BMPR2:ALK3‐Fc (Figure S7B, Supporting Information). Surface plasmon resonance (SPR) spectroscopy demonstrated that GDF10 binds to BMPR2:ALK3‐Fc in a dose‐dependent manner (Figure 5G).

By analyzing publicly available RNA‐seq data, we found that the expression levels of *BMPR2* and *ALK3* vary enormously between different tissues and cells (Figure S7D,E, Supporting Information).^[^ ^32^ ^]^ Notably, we found that *BMPR2* is highly expressed in LSECs and HSCs in both mouse and human livers (Figure 5H,I). Next, we performed RNA‐seq analysis of livers from Gdf10KO and WT mice and found that DEGs are enriched in LSECs and HSCs (Figure S7C, Supporting Information). The phenotypic changes of LSECs, fenestration (protective), and capillarization (promotive), can regulate HSC activation.^[^ ^33^, ^34^ ^]^ We found that GDF10 has a minor effect on LSEC phenotype and does not affect LSEC number and distribution in the liver (Figure S7F–H, Supporting Information). Of note, among the TGF‐β superfamily receptors, *ALK3* is highly expressed in HSCs (Figure 5H,I). Moreover, both *BMPR2* and *ALK3* had high transcript abundance in HSCs, including primary mouse and human HSCs, JS1 cells, and LX‐2 cells^[^ ^21^ ^]^ (Figure S7I–L, Supporting Information). Importantly, RNA‐seq analysis showed that HSCs are the primary effector cells for ECM deposition in the liver (Figure S7C, Supporting Information). CellChat analysis revealed that HSCs serve as the prime target cells for GDF10 (Figure S7M, Supporting Information). Taken together, GDF10 may exert its antifibrotic function mainly by targeting HSCs, and BMPR2/ALK3 is the potential receptor for GDF10.

### 2.6 GDF10 Inhibits HSC Activation via the BMPR2/ALK3‐SMAD1/5/8‐SMAD7 Signaling Pathway

**Figure 6:**
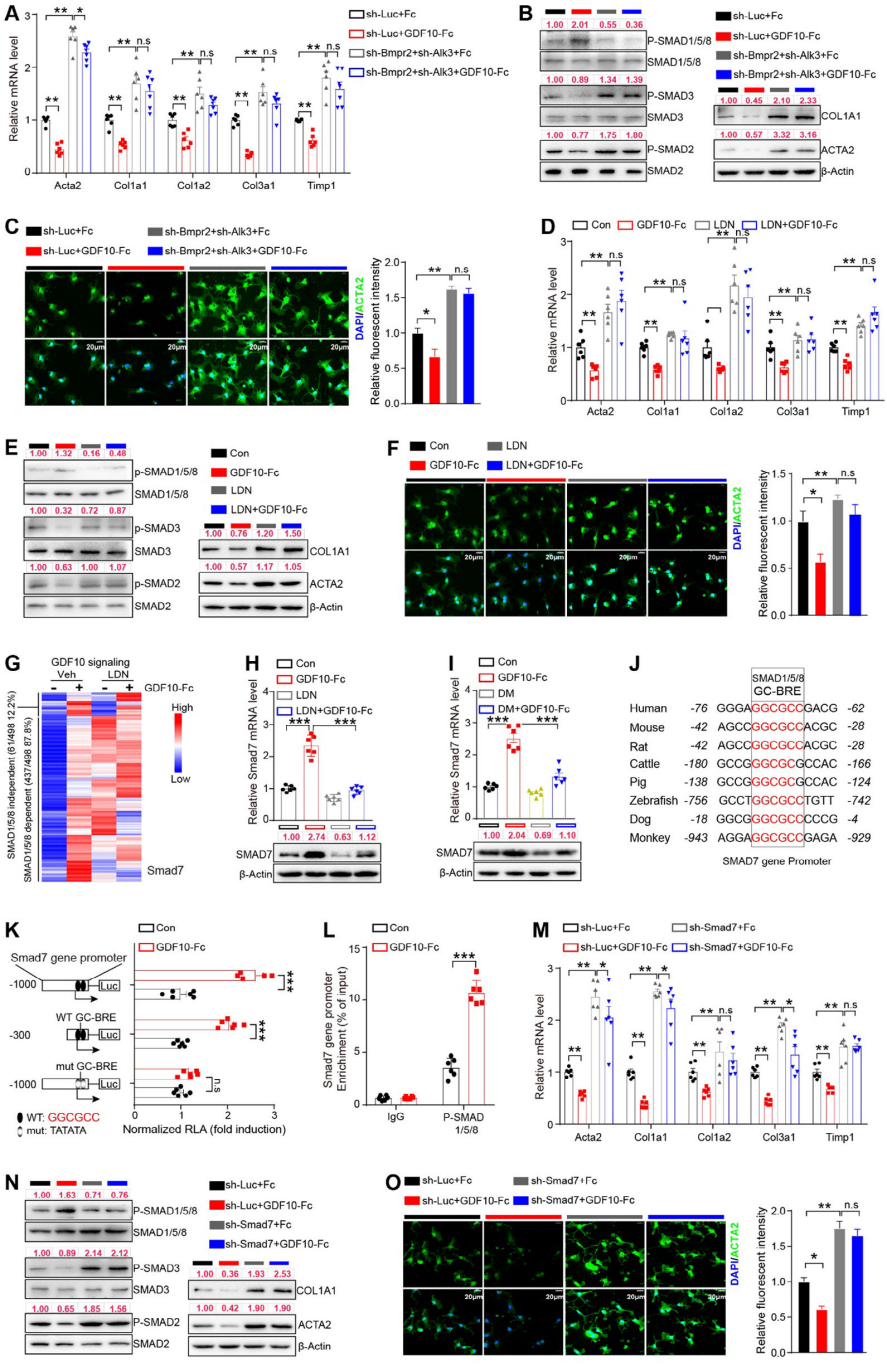
GDF10 inhibits HSC activation via the BMPR2/ALK3‐SMAD1/5/8‐SMAD7 signaling pathway. A,B) qPCR (A) (*n* = 6) and Western blot (B) analysis of indicated genes in the primary mouse HSCs infected with LV‐sh‐Luc or LV‐sh‐Bmpr2 and LV‐sh‐Alk3 for 24 h and then treated with TGF‐β1 plus Fc or GDF10‐Fc for another 24 h. C) IF staining analysis of ACTA2 expression in HSCs treated as in (A), scale bars, 20 µm (*n* = 3). D, E) qPCR (*n* = 6) (D) and Western blot (E) analysis of indicated genes in the HSCs treated with TGF‐β1 plus GDF10‐Fc and/or LDN for 24 h. F) IF analysis of ACTA2 stating in HSCs treated as in (D). G) Heat map shows the indicated genes in JS1 cells treated with TGF‐β1 plus LDN and/or GDF10‐Fc for 24 h. H) qPCR (top) (*n* = 6) and Western blot (bottom) analysis of SMAD7 mRNA and protein levels in HSCs treated as in (G). I) qPCR (top) (*n* = 6) and Western blot (bottom) analysis of SMAD7 mRNA and protein levels in HSCs treated with TGF‐β1 plus DM and/or GDF10‐Fc for 24 h. J) Alignment of the *SMAD7* promoter regions containing the GC‐BRE sites (GGCGCC) in the indicated species. K) Luciferase reporter gene assay in JS1 cells transfected with the indicated plasmids and then treated with Fc or GDF10‐Fc. L) ChIP assay showing the recruitment of phosphorylated SMAD1/5/8 to the *Smad7* gene promoter in JS1 cells (*n* = 3). M,N) qPCR (M) (*n* = 6) and Western blot (N) analysis of indicated genes in the primary mouse HSCs infected with LV‐sh‐Luc or LV‐sh‐Smad7 for 24 h, then treated with TGF‐β1 plus Fc or GDF10‐Fc for another 24 h. O) IF staining analysis of ACTA2 expression in HSCs treated as in (M), scale bars, 20 µm (*n* = 3). Data are mean ± SEM. *p < 0.05, **p < 0.01, ***p < 0.001 by the two‐tailed Student's *t*‐test (K,L), one‐way ANOVA (C,F,H,I,O), or two‐way ANOVA (A,D,M).

To determine whether BMPR2/ALK3 mediates the inhibitory effect of GDF10 on HSC activation, we depleted *Bmpr2* and/or *Alk3* in primary mouse HSCs (Figure S8A,B, Supporting Information) and treated these cells with Fc or GDF10‐Fc. As a result, GDF10‐Fc treatment could not inhibit the expression of HSC activation markers in *Bmpr2*‐ and/or *Alk3*‐depleted HSCs (**Figure** **6**A–C; Figure S8C,D, Supporting Information). ALK3 receptor propagates the signal intracellularly by phosphorylating SMAD1/5/8.^[^ ^28^ ^]^ As expected, GDF10‐Fc treatment enhanced SMAD1/5/8 phosphorylation in primary mouse HSCs, but this enhancement was abolished upon the depletion of *Bmpr2*/*Alk3* (Figure 6B). Furthermore, SMAD1/5/8 signaling antagonist LDN‐193189 (LDN) eliminated the inhibition of GDF10 on the expression of HSC activation markers (Figure 6D–F). TGF‐β‐SMAD2/3 is the canonical signaling involved in HSC activation.^[^ ^2^ ^]^ Of note, GDF10‐Fc treatment suppressed SMAD2/3 phosphorylation in primary mouse HSCs. This suppression disappeared in *Bmpr2*/*Alk3*‐depleted HSCs and LDN‐treated HSCs (Figure 6B,E). These results suggest that GDF10 inhibition of HSC activation depends on the BMPR2/ALK3‐SMAD1/5/8 signaling pathway.

To investigate how GDF10‐BMPR2/ALK3‐SMAD1/5/8 signaling pathway regulates HSC activation, we treated JS1 cells with LDN and/or GDF10‐Fc for 24 h and then performed RNA‐seq analysis. The upregulation of 87.8% GDF10‐response genes was abolished by LDN, further confirming that SMAD1/5/8 is essential for the impact of GDF10 on HSCs (Figure 6G). Among these GDF10‐response genes, *Smad7* caught our attention because SMAD7 can repress TGF‐β signaling by blocking SMAD2/3 phosphorylation^[^ ^35^ ^]^ (Figure 6G). We further validated that SMAD1/5/8 signaling antagonists, LDN and dorsomorphin (DM), treatment prevented GDF10‐stimulated SMAD7 expression in primary mouse HSCs (Figure 6H,I). ChIP‐seq analysis (GSE104682) showed that the promoter upstream of *SMAD7* contains SMAD1/5 binding sites (Figure S8E, Supporting Information).^[^ ^36^ ^]^ Moreover, we identified a conserved SMAD1/5/8 binding motif, GC‐BRE sites (GGCGCC),^[^ ^37^ ^]^ in the promoter of *SMAD7* (Figure 6J). Our promoter deletion and mutation assays confirmed that the GC‐BRE sites mediate the induction of *Smad7* gene expression in response to GDF10 stimulation (Figure 6K). Additionally, our ChIP assays confirmed that phosphorylated SMAD1/5/8 proteins bind to the GC‐BRE sites in the *Smad7* gene promoter region, and GDF10‐Fc treatment enhances this binding efficiency (Figure 6L). Of note, knockdown of *Bmpr2*/*Alk3* also counteracted the stimulatory effect of GDF10 on SMAD7 expression (Figure S8F, Supporting Information). These results indicate that GDF10 upregulates SMAD7 through BMPR2/ALK3‐SMAD1/5/8 signaling pathway.

To determine whether SMAD7 mediates the inhibitory effect of GDF10 on HSC activation, we depleted SMAD7 in primary mouse HSCs (Figure S8G, Supporting Information) and treated these cells with Fc or GDF10‐Fc. Without SMAD7, GDF10 could not suppress the expression of HSC activation markers (Figure 6M–O). Collectively, these results suggest that GDF10 inhibits HSC activation through the BMPR2/ALK3‐SMAD1/5/8‐SMAD7 signaling pathway (Figure S8H, Supporting Information).

### 2.7 Recombinant BMPR2:ALK3‐Fc Protein Blocks the Antifibrotic Effect of GDF10

**Figure 7:**
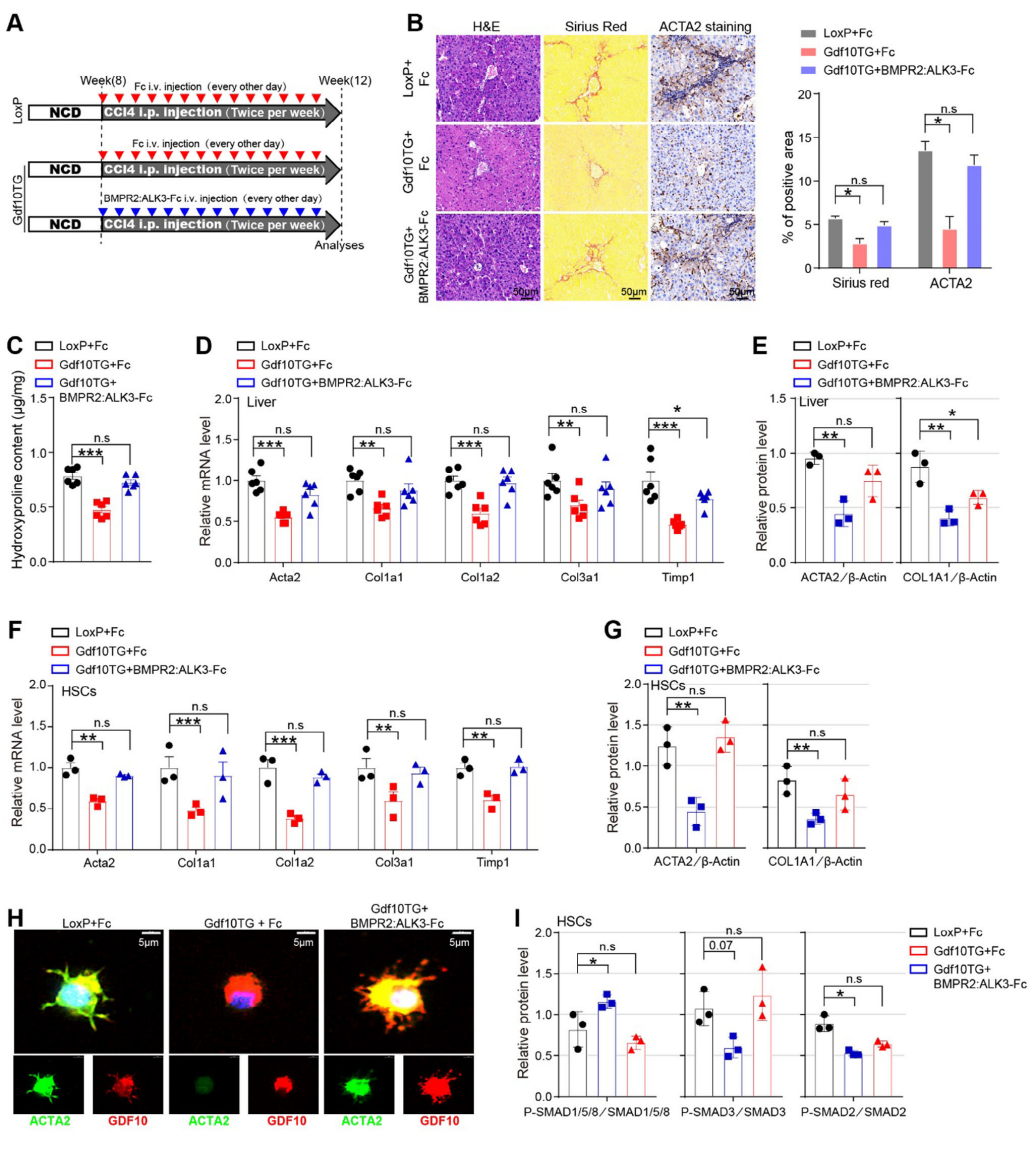
BMPR2:ALK3‐Fc prevents the antifibrotic effect of GDF10 in the liver. A) Schematic drawing of the experimental procedure in 3‐month‐old male LoxP and Gdf10TG mice. B) Representative images of H&E, Sirius Red, and ACTA2 IHC staining, scale bars, 50 µm (*n* = 3). C) Hydroxyproline assay analysis of the total liver collagen of mice treated as in (A) (*n* = 6). D,E) qPCR (D) (*n* = 6) and Western blot (E) analysis of indicated genes in the liver of mice treated as in (A). F,G) qPCR (F) (*n* = 6) and Western blot (G) analysis of indicated genes in the HSCs from mice treated as in (A) (*n* = 3). H,I) IF staining (H) and Western blot (I) analysis of indicated protein in the HSCs from mice treated as in (A), scale bars, 5 µm. Data are mean ± SEM. *p < 0.05, **p < 0.01, ***p < 0.001 by the one‐way ANOVA (C,E,G,I) or two‐way ANOVA (D,F).

To probe the involvement of BMPR2/ALK3 in the antifibrotic effect of GDF10, CCl4‐injected male Gdf10TG and LoxP mice were treated with recombinant BMPR2:ALK3‐Fc proteins or Fc proteins (**Figure** **7**A). BMPR2:ALK3‐Fc treatment reversed the reduction of Sirius Red staining (Figure 7B), hydroxyproline content (Figure 7C), and expression of fibrogenic genes (Figure 7D,E; Figure S9A, Supporting Information) in the livers of Gdf10TG mice. We isolated HSCs from these mice and found that BMPR2:ALK3‐Fc treatment intercepted GDF10‐inhibited expression of HSC activation marker genes (Figure 7F–H; Figure S9A, Supporting Information). Meanwhile, BMPR2:ALK3‐Fc treatment reversed the activation of SMAD1/5/8 and the suppression of SMAD2/3 in HSCs of Gdf10TG mice (Figure 7I; Figure S9A, Supporting Information). However, GDF10 did not impact SMAD1/5/8 signaling or *Acta2* and *Col1a1* expression in HCs and MACs, with or without BMPR2:ALK3‐Fc (Figure S9B–E, Supporting Information). Therefore, BMPR2:ALK3‐Fc predominantly blocks GDF10 signaling transduction in HSCs and intercepts the antifibrotic effects of GDF10.

### 2.8 Therapeutic Administration of Recombinant GDF10 Protein Ameliorates Hepatic Fibrosis

**Figure 8:**
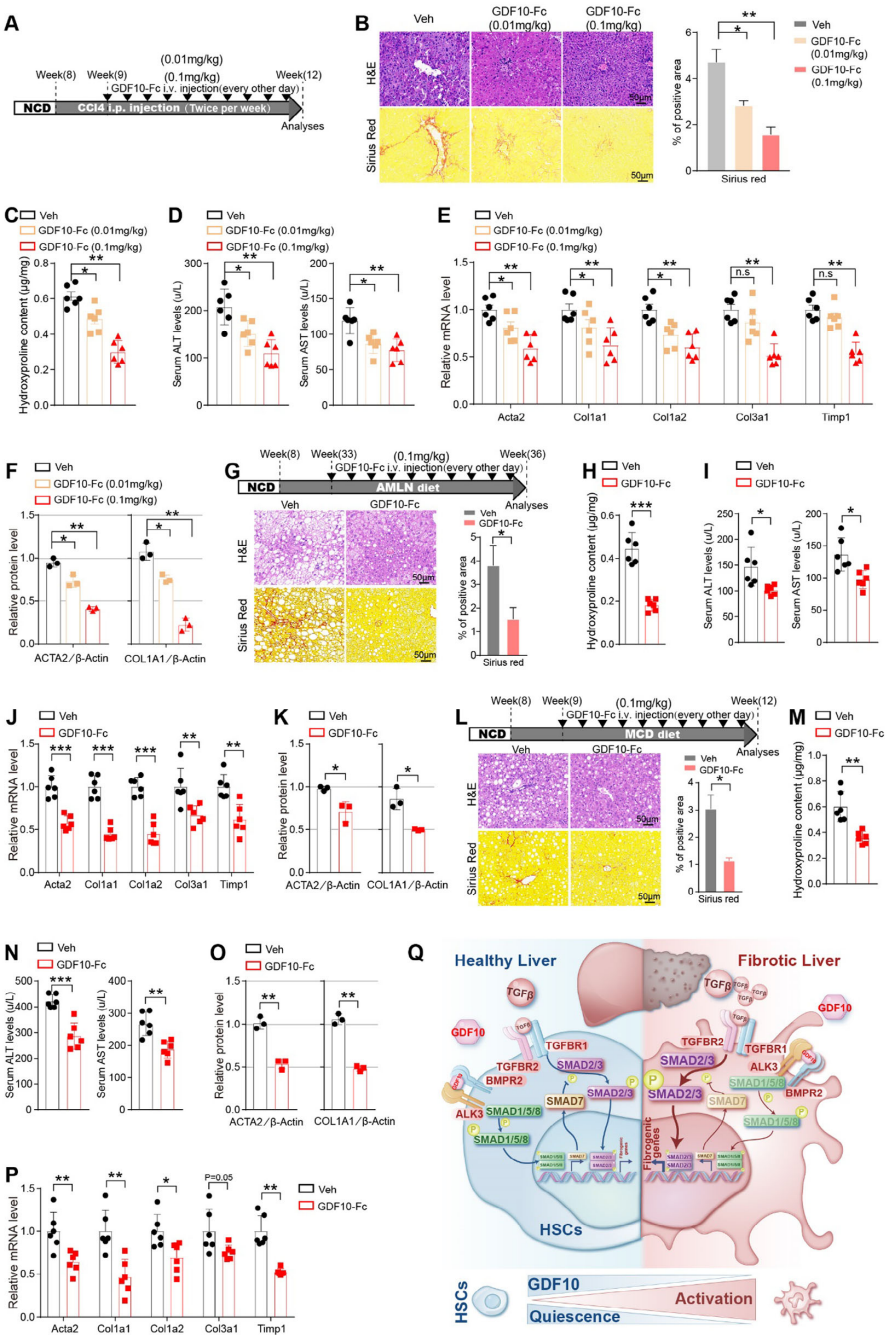
Therapeutic administration of GDF10‐Fc ameliorates hepatic fibrosis. A) Schematic drawing of the experimental procedure. B) Representative images of H&E and Sirius Red staining of liver from mice treated as in (A), scale bars, 50 µm (*n* = 3). C,D) Measurement of the total liver collagen content by hydroxyproline assay (C) and serum ALT and AST levels (D) in the mice treated as in (A) (*n* = 6). E, F) qPCR (E) (*n* = 6) and Western blot (F) analysis of indicated genes in the liver of mice treated as in (A). G) Schematic drawing of the experimental procedure and representative images of H&E and Sirius Red staining of liver from AMLN diet‐induced fibrosis, scale bars, 50 µm (*n* = 3). H,I) Measurement of the total liver collagen content by hydroxyproline assay (H) and serum ALT and AST levels (I) of the mice treated as in (G) (*n* = 6). J,K) qPCR (J) (*n* = 6) and Western blot (K) analysis of indicated genes in the liver of mice treated as in (G). L) Schematic drawing of the experimental procedure and representative images of H&E and Sirius Red staining of liver from the MCD diet‐induced fibrosis, scale bars, 50 µm (*n* = 3). M,N) Measurement of the total liver collagen content by hydroxyproline assay (M) and serum ALT and AST levels (N) of the mice treated as in (L) (*n* = 6). O, P) Western blot (O) and qPCR (P) (*n* = 6) analysis of indicated genes in the liver of mice treated as in (L). Q) The proposed model of GDF10 prevents liver fibrosis. Data are mean ± SEM. *p < 0.05, **p < 0.01, ***p < 0.001 by the two‐tailed Student's *t*‐test (G–P), one‐way ANOVA (B,C,D,F), or two‐way ANOVA (E).

We next evaluated the therapeutic potential of GDF10 in liver fibrosis. CCl4‐induced fibrosis model mice were treated with vehicle or GDF10‐Fc (0.01 mg kg^−1^ or 0.1 mg kg^−1^) (**Figure** **8**A). As expected, GDF10‐Fc treatment reduced liver Sirius Red staining, hydroxyproline content, and fibrogenic gene expression (Figure 8B–F; Figure S10A, Supporting Information). GDF10‐Fc‐treated mice had decreased serum ALT and AST compared to the control mice. Furthermore, GDF10‐Fc treatment effectively inhibited HSC activation in mice (Figure S10B, Supporting Information). These results indicate a promising therapeutic effect of GDF10 in the treatment of liver fibrosis.

We also examined the therapeutic potential of GDF10 in AMLN diet‐induced and MCD diet‐induced mouse liver fibrosis. Similarly, we observed that GDF10‐Fc treatment reduced liver Sirius Red staining, hydroxyproline content, and fibrogenic gene expression and decreased serum ALT and AST levels in AMLN diet‐fed and MCD diet‐fed mice (Figure 8G–P; Figure S10C,D, Supporting Information).

In conclusion, the results demonstrate that administration of GDF10 effectively attenuates HSC activation and shows promising outcomes in the reversal of mouse liver fibrosis with different etiologies.

## 3 Discussion

Liver fibrosis prevalence is increasing globally, thus burdening public health. Currently, no specific treatment exists for liver fibrosis. In this study, we identified GDF10 as a critical regulator of HSC activation and a promising therapeutic target for liver fibrosis. GDF10 was specifically expressed in HSCs in the liver, and its expression was downregulated in activated HSCs. Loss of GDF10 promoted HSC activation and liver fibrosis, whereas gain of GDF10 alleviated this pathological condition. Consistently, recombinant GDF10 protein treatment ameliorated CCl4‐induced, AMLN diet‐induced, and MCD diet‐induced liver fibrosis. Thus, pharmacologically targeting GDF10 could be explored in the treatment of liver fibrosis.

Type I (ALK1‐7) and type II (BMPR2, TGFBR2, ACVR2A, ACVR2B, and AMHR2 (only in humans)) serine/threonine kinase receptors facilitate signal transduction of TGF‐β superfamily ligands by activating R‐SMADs.^[^ ^28^ ^]^ As a member of TGF‐β superfamily ligands, GDF10 has been reported to activate distinct R‐SMADs in different cells to achieve its biological functions.^[^ ^8^, ^10^, ^11^, ^38^ ^]^ The studies showed that GDF10 may have distinct functions in different cell types and potentially signal through diverse TGF‐β superfamily receptors. A recent study demonstrated that GDF10 signals through SMAD3, thereby inhibiting PPARγ activity in HCs, resulting in reduced lipid droplet accumulation. Consequently, *Gdf10* knockout mice exhibit increased steatosis and fibrosis under a high‐fat diet.^[^ ^12^ ^]^ PPARγ has proven to inhibit HSC activation, and its activity is diminished in activated HSCs.^[^ ^2^, ^39^ ^]^ These studies also suggest that the function of GDF10 may exhibit cell type‐specific characteristics. A previous study has reported that GDF10 binds to ALK5.^[^ ^29^ ^]^ Upon comparing the GDF10‐binding ability of all TGF‐β superfamily receptors, we found that BMPR2 has higher GDF10 recognition potential than others. Of note, we found that the expression profiles of TGF‐β superfamily receptors vary in different tissues and cells. Therefore, the diverse biological functions of GDF10 are likely attributed to the differing expression patterns of TGF‐β superfamily receptors within tissues and cells.

Some ligands of the TGF‐β superfamily exhibit relatively low affinity for type I or type II receptors alone; however, their binding affinity is significantly increased when both types of receptors are present.^[^ ^31^ ^]^ This study revealed that ALK3, unlike other type I receptors, greatly enhances the GDF10 recognition potential of BMPR2. Our molecular analysis further suggests that BMPR2/ALK3 heterodimeric functions as a receptor for GDF10. Additionally, BMPR2/ALK3 heterodimer was essential for GDF10 to inhibit liver fibrosis. Previous studies have revealed that BMPR2 and ALK3 signaling protects against kidney, pancreatic, and lung fibrosis,^[^ ^40^ ^]^ supporting our conclusions. GDF10 has been reported to signal via activating SMAD1/5/8 in myofibroblasts and cardiomyocytes, but its receptor is unknown.^[^ ^10^, ^41^ ^]^ We speculate that the BMPR2/ALK3 heterodimer could potentially act as a receptor for GDF10 in myotubes and cardiomyocytes.

HSC activation is regulated by core and regulatory pathways.^[^ ^2^, ^42^ ^]^ Core pathways are involved in fibrosis across tissues and disease contexts, while regulatory pathways are more tissue‐specific.^[^ ^2^ ^]^ Although targeting core pathways may have a broader impact across organs, it carries a higher risk of off‐target effects.^[^ ^2^ ^]^ TGF‐β is recognized as a universal driver of fibrosis. Systemic inhibition of TGF‐β signaling can stimulate cell proliferation and enhance tumor response, thereby limiting its clinical application.^[^ ^43^ ^]^ Therefore, targeting HSC‐specific factors that regulate TGF‐β signaling presents a potential avenue for effective and safe treatments of liver fibrosis. Due to the higher expression of *Alk3* in HSCs than in other liver cells (Figure S6C), GDF10 does not affect the SMAD1/5/8 signaling in HCs and MACs. Additionally, global *Gdf10* knockout does not result in any discernible effects on tissue development.^[^ ^7^ ^]^ Thus, GDF10 could be an HSC‐specific drug target for liver fibrosis, as it mainly inhibits TGF‐β signaling in HSCs but not in other cells.

There are several limitations to our study. For instance, we employed global *Gdf10* knockout mice for in vivo GDF10 loss‐of‐function studies. However, the optimal approach would be to specifically knock out *Gdf10* in HSCs while inhibiting circulating GDF10 get to HSCs. Multiple liver cell types express BMPR2 and are involved in the regulation of HSC activation.^[^ ^33^ ^]^ Thus, the autocrine effect of GDF10 in inhibiting HSC activation may be only one of the mechanisms underlying its antifibrotic effects. Future studies need to use cell‐specific conditional BMPR2 knockout mice to investigate the signaling mechanism of GDF10 in vivo. In addition, other BMPs may signal through BMPR2 and also need to be considered. Finally, whether the effect of GDF10 on mouse HSC biology extends to human HSCs in vivo deserves further study.

In summary, autocrine GDF10 activates BMPR2/ALK3‐SMAD1/5/8‐SMAD7 pathway to counteract the TGF‐β‐SMAD2/3 pathway in HSCs, thereby inhibiting HSC activation and preventing liver fibrosis (Figure 8Q). This study highlights the profound therapeutic potential of GDF10 in patients with liver fibrosis.

## 4 Experimental Section

### Study Design

The animals were weighed before the experiment to eliminate this potential confounding variable. Animals were then randomly assigned to either the experimental or control groups. Data analysis was performed in a blinded fashion, including all animals, without any exclusions. Until the end of data collection, group assignments and other information were kept blind to the investigators. Cell experiments were not blinded. Most experiments were independently replicated three or more times, with the sample size (n) for each experimental group or condition given in the figure legends.

### Animal

LoxP mice were generated using the CRISPR/Cas9 system to insert a LoxP‐STOP‐LoxP‐Gdf10 cassette into the mouse Rosa26 locus. Subsequently, LoxP mice were crossed with Lrat‐Cre transgenic mice to generate liver‐specific *Gdf10* transgenic mice (Gdf10TG); Cre‐negative littermates were employed as controls. *Gdf10* knockout (Gdf10KO) mice were generated by using the CRISPR/Cas9 to delete exon 2 of *Gdf10* by co‐injecting Cas9 and gRNA into fertilized eggs; WT littermates were employed as controls. All genotypes were generated on a pure C57BL/6J background and genotyped by PCR. The Institutional Animal Care and Use Committee of Tianjin Medical University approved all study protocols involving animals.

### Liver Fibrosis Models

For CCl4‐induced liver fibrosis model, mice were intraperitoneally injected with 0.5 mL kg^−1^ CCl4 (dissolved in a 1:3 ratio with corn oil) twice weekly for 4 weeks. For BDL‐induced liver fibrosis model, mice were anesthetized and subjected to midline laparotomy. The common bile duct was ligated twice with silk sutures and the abdomen closed. The control mice underwent a similar procedure, but the common bile duct was not ligated. For the metabolic‐induced liver fibrosis model, mice were fed with the AMLN diet (D09100301, Research Diets Inc.) for 28 weeks starting at 3 months of age, or the MCD diet (A02082002B, Research Diets Inc.) for 4 weeks starting at 2 months of age.

### Cell Culture

Human hepatic stellate cell line LX‐2, murine hepatic stellate cell line JS1, and human embryonic kidney 293T (HEK293T) cells were maintained in DMEM (Gibco, 11995081) supplemented with 10% fetal bovine serum (Gibco, 10099).

### Isolation of Primary Cells in the Liver

Using a previously published protocol, primary mouse cells were isolated from the liver.^[^ ^44^ ^]^ The liver tissue underwent digestion through retrograde stepwise perfusion using solutions containing collagenase (Sigma‐Aldrich, 11213865001) and pronase (Sigma‐Aldrich, 11459643001). Subsequently, the liver cell suspension was centrifuged at 50 × *g* (4 °C) for 5 min to separate hepatocytes from nonparenchymal cells (NPCs). The isolated hepatocytes were then plated on collagen‐coated dishes and cultured in hepatocyte maintenance medium. HSCs were then isolated from NPCs using a Nycodenz density gradient centrifugation method. The gradient was centrifuged at 1380 × *g* for 17 min at 4 °C without brakes. Subsequently, the purified HSCs were cultured in DMEM supplemented with 10% fetal bovine serum. LSECs and MACs were purified from NPCs using the OptiPrep density gradient centrifugation method. The OptiPrep gradient was subjected to centrifugation at 1400 × *g* for 15 min without brake, resulting in the isolation of a mixed suspension of LSECs and MACs. In contrast to LSECs, MACs exhibit rapid adherence to cell culture dishes, which effectively separates them from LSECs. Subsequently, the isolated LSECs and MACs were resuspended in an LSEC medium and incubated at 37 °C for 1 h. MACs were found to be the most adherent cells, whereas LSECs were predominantly nonadherent. Mixed male and female mice were used for all primary cell experiments.

### Construction and Expression of Recombinant BMPR3:ALK3‐Fc

BMPR2:ALK3‐Fc was generated by ligation of the extracellular domains (ECDs) of mouse BMPR2 (aa 27–150) or mouse ALK3 (aa 24–152) to a modified human IgG1 Fc domain (KiH heterodimeric Fc variants). The 2 gene sequence was synthesized by Tsingke Biotechnology. and then cloned into the pcDNA3.1 vector. The two gene sequences were synthesized by Tsingke Biotechnology. and then cloned into the pcDNA3.1 vector. The two plasmids were co‐expressed in HEK293T cells, resulting in the soluble expression of the BMPR2:ALK3‐Fc fusion protein. Protein A affinity chromatography was then employed for protein purification. GDF10‐Fc was purchased from Sino Biological (Cat: 50165‐M01H). Briefly, the DNA sequence encoding the mature form of mouse GDF10 (NP_665684.2) (Gln 367‐Arg 476) was fused with the Fc region of human IgG1 at the N‐terminus. The expressing plasmid was expressed in HEK293T cells, resulting in the soluble expression of the GDF10‐Fc fusion protein.

### Quantitative PCR

Total RNA was extracted using Trizol reagent (Invitrogen), followed by cDNA synthesis using the Applied Biosystems cDNA Reverse Transcription Kit. Subsequently, qPCR analysis was performed using the SYBR green‐based assay on the Roche LightCycler 96 system. The relative levels of expression of the mRNA were determined after normalization to the expression of 36B4. Primer sequences for qPCR are shown in Table S1, Supporting Information.

### Construction of Recombinant Lentivirus

To knock down the expression of *Gdf10*, *Bmpr2*, *Alk3*, and *Smad7*, lentivirus‐based short hairpin RNA (shRNA) expression constructs were created using the pLKO.1 vector (Addgene). Small interfering RNA sequence for *Gdf10* is CCACATGCCCTATATCCTT;^[^ ^30^ ^]^ *Bmpr2* is AACGCAACCTGTCACATAATA;^[^ ^45^ ^]^ *Alk3* is GCTATTAATAACACATGCATA;^[^ ^45^ ^]^ *Smad7* is GCTATTAATAACACATGCATA.^[^ ^45^ ^]^ Next, *Gdf10* cDNA was cloned into the pCDH‐CMV‐MCS‐EF1‐puro lentiviral vector (Addgene) to construct a *Gdf10* overexpression lentivirus. Lentiviral particles were produced by co‐transfecting the shRNA plasmid or overexpression vector targeting a specific gene, along with the envelope vector pMD2.G and packaging vector psPAX2 and in 293T cells.

### Western Blot Analysis

Cells and tissues were lysed in the lysis buffer (140 mm NaCl, 20 mm Tris‐Cl pH 7.5, 10 mm NaF, 1 mm CaCl2 and MgCl2, 10% glycerol, 1% NP‐40, and 2 mm Na‐vanadate) supplemented with protease inhibitor cocktail, 1 mm phenylmethylsulfonyl fluoride (PMSF), and phosphatase inhibitor cocktail. The cell lysates were gently vortexed for 60 minutes and then centrifuged at 13000 × g for 15 minutes at 4 °C. The BCA assay was used for protein quantification. 20–50 µg of protein lysate was separated by SDS‐PAGE and then transferred to the PVDF membranes (Millipore). After wet transfer, the PVDF membranes were blocked for 1 hour in Tris‐buffered saline containing 5% nonfat dry milk and 0.1% Tween‐20 at room temperature. The primary antibody was incubated overnight at 4 °C, followed by the secondary antibody for 60 minutes at room temperature. For Western blot analysis, the following primary antibodies were employed: β‐Actin (AC026; ABclonal), GDF10 (ab235005; Abcam), ACTA2 (A17910; ABclonal), COL1A1 (A22089; ABclonal), phospho‐SMAD2‐S467 (AP0269; ABclonal), SMAD2 (A19114; ABclonal), phospho‐SMAD3‐S423/S425 (AP1263; ABclonal), SMAD3 (A19115; ABclonal), phospho‐SMAD1/5/8 (#13 820; Cell Signaling Technology), SMAD15/8 (A17439; ABclonal), BMPR2 (sc‐393304; Santa Cruz Biotechnology), ALK3 (sc‐134285; Santa Cruz Biotechnology), SMAD7 (sc‐365846; Santa Cruz Biotechnology). Desmin (GB12075; Servicebio).

### Chromatin Immunoprecipitation

Mouse JS1 cells were homogenized and treated with 1% formaldehyde for 10 minutes at room temperature and lysed with ChIP cell lysis buffer (10 mm Tris‐HCl, pH 8.0, 3 mm MgCl2, 10 mm NaCl, 0.5% NP‐40, 1 mm PMSF, and protease inhibitor cocktail) and ChIP nuclear lysis buffer (50 mm Tris‐HCl, pH 8.0, 1% SDS, 5 mm EDTA, 1 mm PMSF, and protease inhibitor cocktail). Cell lysates were sonicated to shear the chromatin. Equal aliquots of cell lysates were incubated with normal rabbit IgG or anti‐phospho‐SMAD1/5/8 antibody for (#13820; Cell Signaling Technology) overnight at 4 °C. Cell lysates were then incubated with Protein A agarose beads for 4 hours at 4 °C. Phospho‐SMAD1/5/8 and its bound DNA fragments were then immunoprecipitated with protein A agarose beads. Non‐specifically bound DNA and proteins were washed away with a washing buffer to ensure that only the specific phospho‐SMAD1/5/8‐DNA complexes were isolated. qPCR analysis was performed to detect the indicated locus. Primer sequences are shown in Supporting Table S2.

### Co‐Immunoprecipitation

Cells were lysed with NP‐40 buffer (150 mm sodium chloride, 50 mm Tris, pH 7.5, 0.25% sodium deoxycholate, 1 mm ethylenediaminetetraacetic acid) supplemented with protease inhibitor cocktail and 1 mm PMSF. Cell lysates were incubated with normal IgG overnight at 4 °C, anti‐Flag (MBL BEIJING BIOTECH CO., LTD, catalog no. M185‐3L), or anti‐His antibody (MBL BEIJING BIOTECH CO., LTD, catalog no. PM032). Cells lysates were then incubated with Protein A agarose beads for 4 hours at 4 °C. The beads were collected and washed three times with lysis buffer to remove non‐specific proteins, after which immunoblotting (IB) was performed using antibodies specific to the indicated proteins. For BMPR2 and ALK3 interacted with GDF10‐Fc, 500 µg protein was immunoprecipitated with Flag‐conjugated beads overnight at 4 °C. Anti‐Flag and anti‐human Fc antibodies (ABclonal, A21291) were then used to perform IB.

### Immunofluorescence

The cells and livers were fixed with 4% paraformaldehyde for 10 minutes at room temperature. The cells and livers were then blocked with 5% BSA in PBS for 1 hour at room temperature, after which they were incubated with primary antibodies (diluted in blocking buffer) overnight at 4 °C or for 1 hour at room temperature. Subsequently, cells and livers were incubated with a secondary antibody conjugated to a fluorophore for 1 hour at room temperature in the dark. Finally, nuclei were stained with DAPI.

### Hydroxyproline Assay and ELISA

The total collagen content of the liver was quantified by measuring the hydroxyproline levels using hydroxyproline detection kits from the Nan Jing Jan Cheng Biochemical Institute in Nanjing, China. The circulating levels of GDF10 were measured using a mouse GDF10 ELISA kit (Wuhan Fine Biotech Co., Ltd.)

### Histology Analysis

The livers were fixed in 10% formalin for 24 h, after which they were dehydrated using a series of ethanol solutions (70%, 80%, 90%, 100%). The livers were then embedded in paraffin wax and stained with Sirius red, hematoxylin and eosin, or immunohistochemistry. The images were taken under a microscope.

### Flow Cytometric Analysis

293 cells were transiently transfected with TGF‐β superfamily receptor cDNA and then incubated with GDF10‐Fc or Fc in the presence of 5% fetal bovine serum at 4 °C for 1 hour. The cells were collected and washed three times with PBS to remove non‐specific proteins, followed by incubation with Alexa Fluor 647 goat anti‐human IgG (H+L) antibody (Thermo Fisher, A‐21445) for 1 hour at 4 °C. The stained cells were then loaded into the flow cytometer.

### Statistical Analysis

Data presented as mean ± standard error of mean (SEM). For pairwise comparisons of genotypes or treatments, a Student's *t*‐test was used. One‐way ANOVA and two‐way ANOVA were used for comparisons between three or more groups. GraphPad Prism and/or Microsoft Excel were used to analyze the data. *p* < 0.05 was considered statistically significant.

## Conflict of Interest

The authors declare no conflict of interest.

## Author Contributions

Y.Z., X.G., Y.L., and Z.C. contributed equally to this work. Y.Z., J.Z., Z.P., and Y.C. designed the research. Y.Z., X.G., Y.L., and Z.C. performed the research. Y.Z., X.Z., W.Q., P.Q., C.D., S.S., J.H., Y.Z., and H.F. acquired data. X.L. and M.L. provided the experimental assistance. Y.Z. analyzed data. Y.C. and Y.Z. wrote the paper. The manuscript has been read and approved by all authors.

## Supporting information



Supporting Information

## Data Availability

The RNA‐seq data are deposited at NCBI under SRA (SRA accession number: PRJNA1229478). All data are available from the corresponding author on reasonable request.
