# Supplementary material for: Autocrine GDF10 Inhibits Hepatic Stellate Cell Activation via BMPR2/ALK3 Receptor to Prevent Liver Fibrosis
Source: Adv Sci (Weinh). 2025 Mar 24;12(19):2500616. doi: 10.1002/advs.202500616 (PMC12097095; doi:10.1002/advs.202500616)
Supplement: Supplementary file 1 — Supporting Information [file ADVS-12-2500616-s001.docx]

Supporting Information

**Autocrine GDF10 inhibits hepatic stellate cell activation via BMPR2/ALK3 receptor to prevent liver fibrosis**

*Yinliang Zhang, Xiaochen Gai, Yuhui Li, Zuoyu Chen, Xi Zhang, Wei Qiao, Ping Qiu, Chunyuan Du, Sufang Sheng, Jingran Hao, Yujie Zhang, Heng Fan, Xiaorong Li, Ming Liu, Jun Zhang*, Zhe Pan*, Yongsheng Chang**


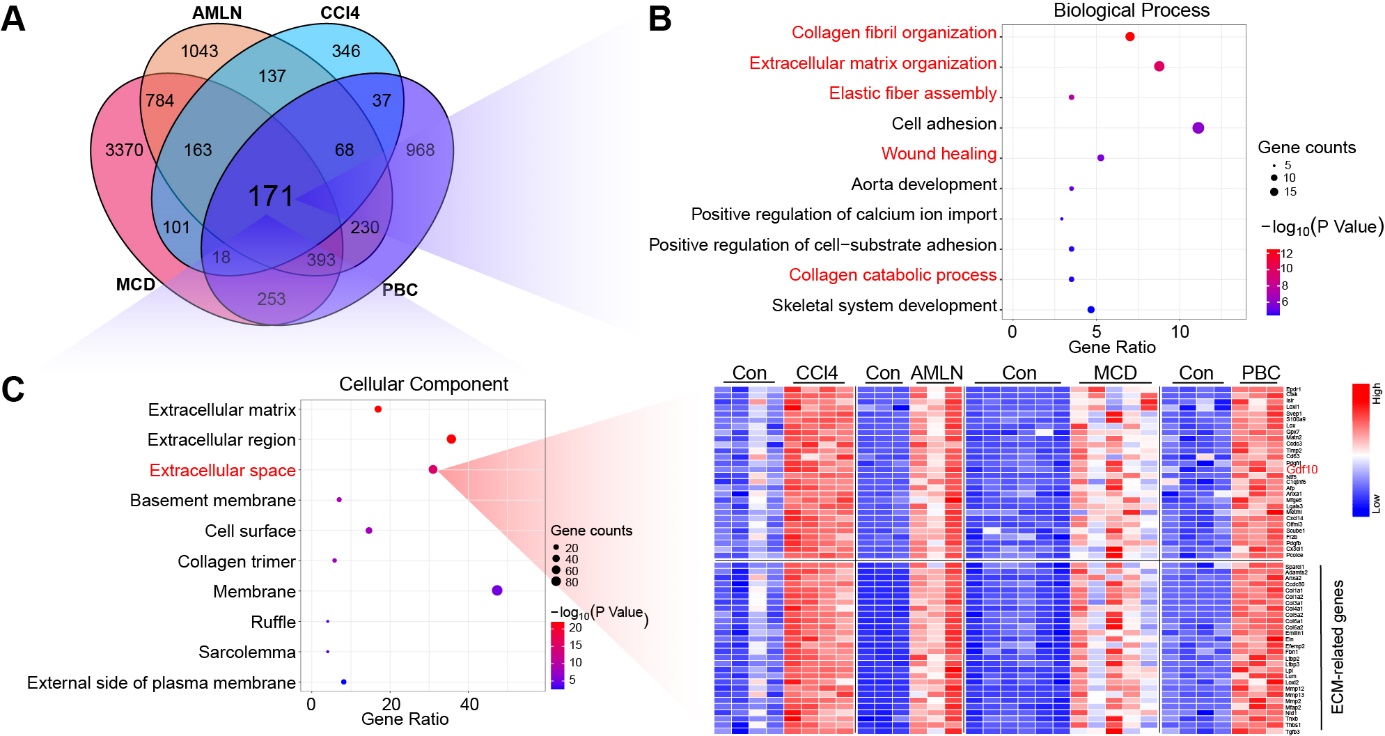
 **Figure S1.** GDF10 is a liver fibrosis-associated cytokine. A) Venn diagrams of differentially expressed genes (DEGs, log2FC > 1 or < −1, FDR < 0.05) identified from CCl4-induced, PBC-induced, MCD diet-induced, and AMLN diet-induced mouse models of liver fibrosis. B, C) GO biological process (B) and cellular component (C) enrichment analysis of the shared DEGs in (A).


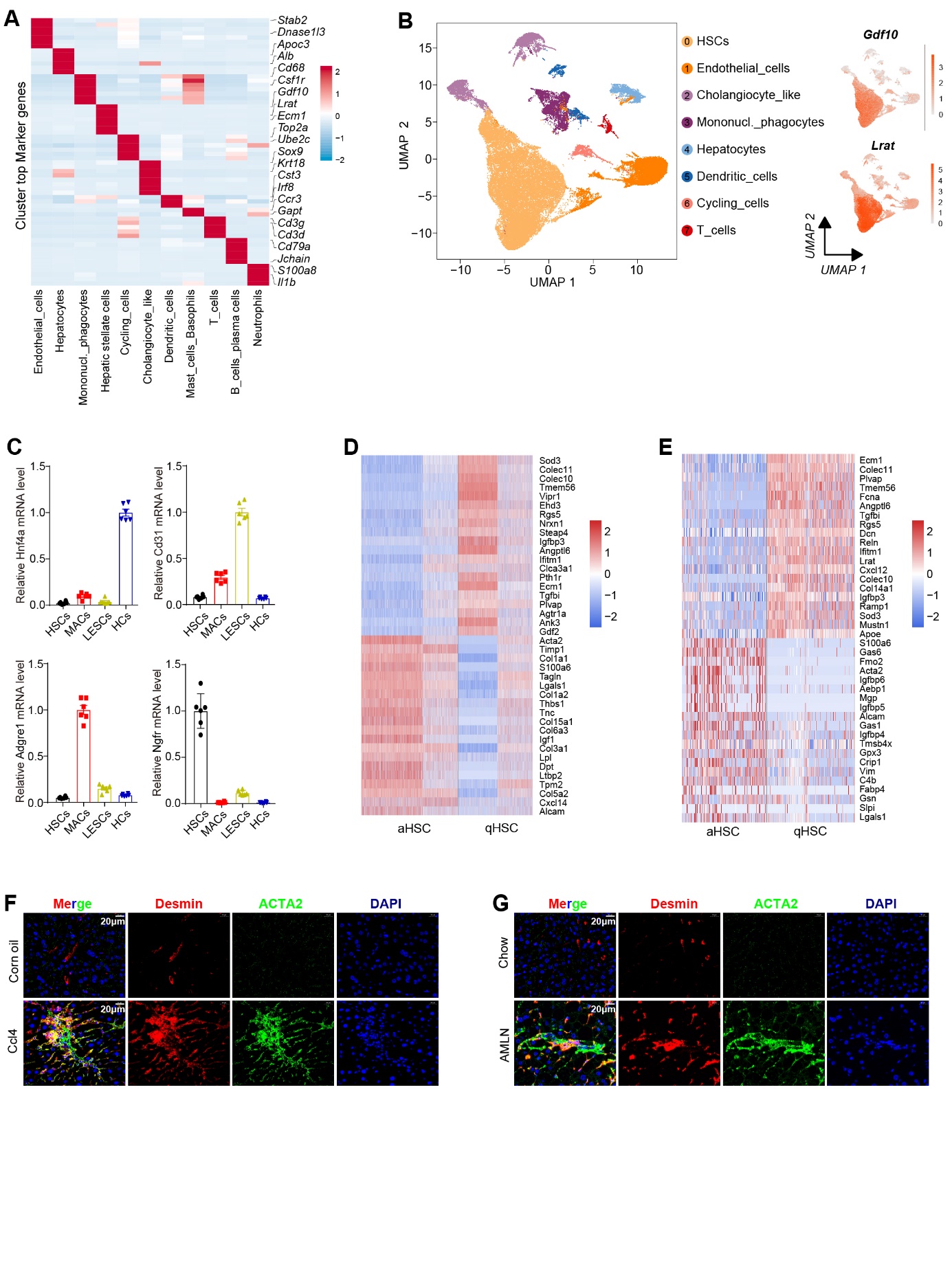
 **Figure S2.** GDF10 expression in the liver and HSC number is increased in fibrotic liver. A) Heatmap showing representative marker gene expression for each cluster, data from GSE218299. B) UMAP visualization of the cell type expresses *Gdf10* in the liver (GSE171904). C) qPCR analysis of *Hnf4a*, *Cd31*, *Adgre1*, and *Ngfr* mRNA levels in HCs, LSECs, MACs, and HSCs from normal liver. D, E) Heat map depicting the top 20 representative genes of qHSC and aHSC in GSE218299 (D) and GSE171904 (E). F, G) IF staining analysis of Desmin and ACTA2 protein levels in the liver, scale bars, 20 µm.


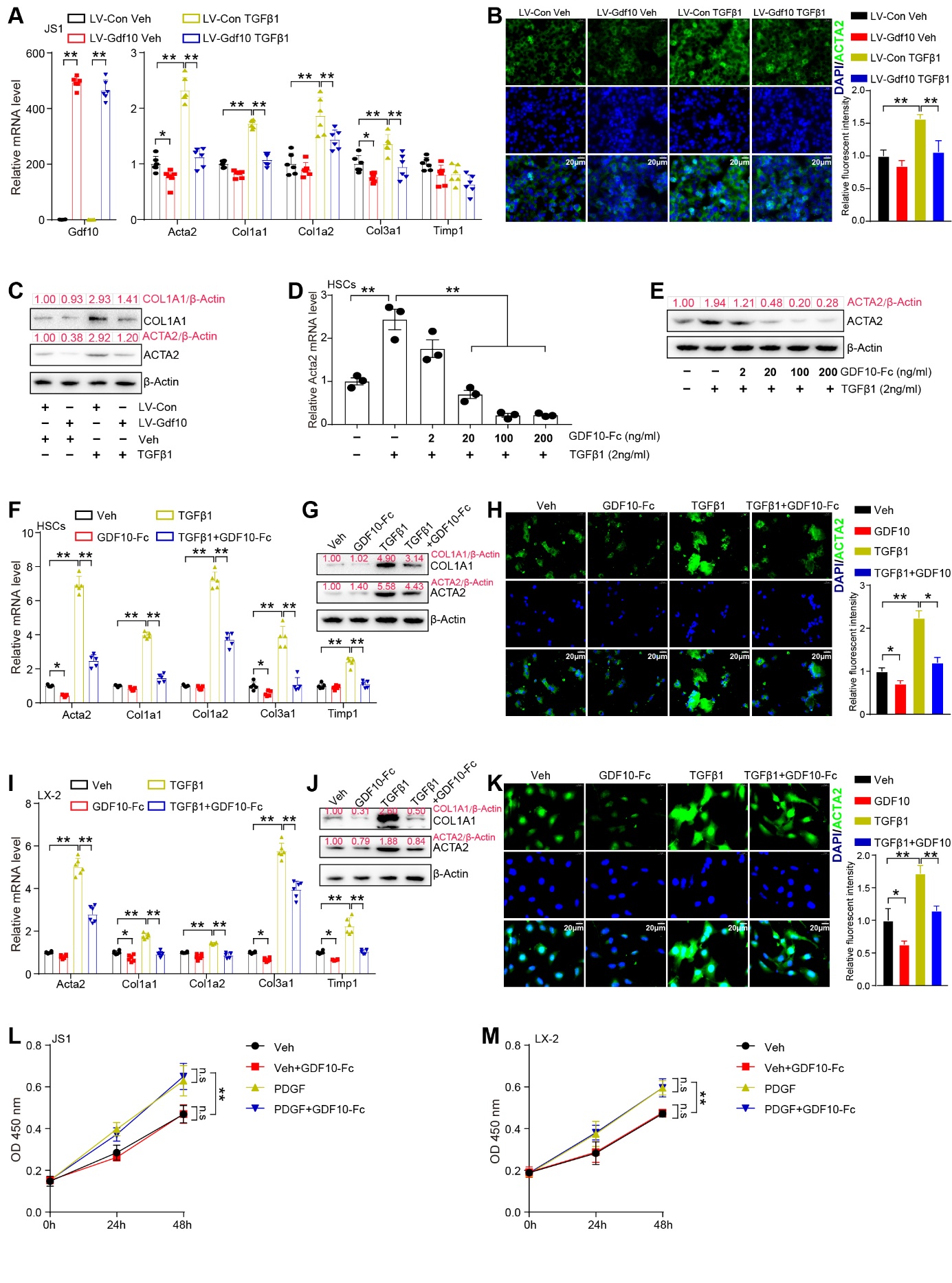
 **Figure S3.** GDF10 represses HSC activation. A) qPCR analysis of indicated genes in mouse HSC cell line (JS1 cells) infected with LV-Con or LV-Gdf10 for 24 h and then treated with TGFβ1 (2 ng/mL) or vehicle for another 24 h (n = 6). B) IF analysis of ACTA2 stating in JS1 cells treated as in (A), the relative fluorescence intensity of ACTA2 is also shown (right) (n = 3). C) Western blot analysis of indicated genes in JS1 cells treated with as in (A). D, E) qPCR (D) (n = 3) and Western blot (E) analysis of ACTA2 expression in primary mouse HSCs treated with TGFβ1 (2 ng/mL) and corresponding concentrations of GDF10-Fc for 24 h. F, G) qPCR (n = 5) (F) and Western blot (G) analysis of indicated genes in primary mouse HSCs treated with TGFβ1 (2 ng/mL) and/or GDF10-Fc (20 ng/mL) for 24 h. H) IF analysis of ACTA2 stating in HSCs treated as in (F), the relative fluorescence intensity of ACTA2 is also shown (right) (n = 3). I, J) qPCR (n = 6) (I) and Western blot (J) analysis of indicated genes in LX-2 cells treated as in (F). K) IF analysis of ACTA2 stating in LX-2 cells treated as in (F), the relative fluorescence intensity of ACTA2 is also shown (right) (n = 3). L, M) Cell Counting Kit-8 assay analysis the proliferation of JS1 cells (a mouse hepatic stellate cell line) (L) and LX-2 cells (M). Data are mean ± SEM. * P < 0.05, ** P < 0.01, *** P < 0.001 by the one-way ANOVA (A (left), B, D, H, K), or two-way ANOVA (A (right), F, I, L, M).


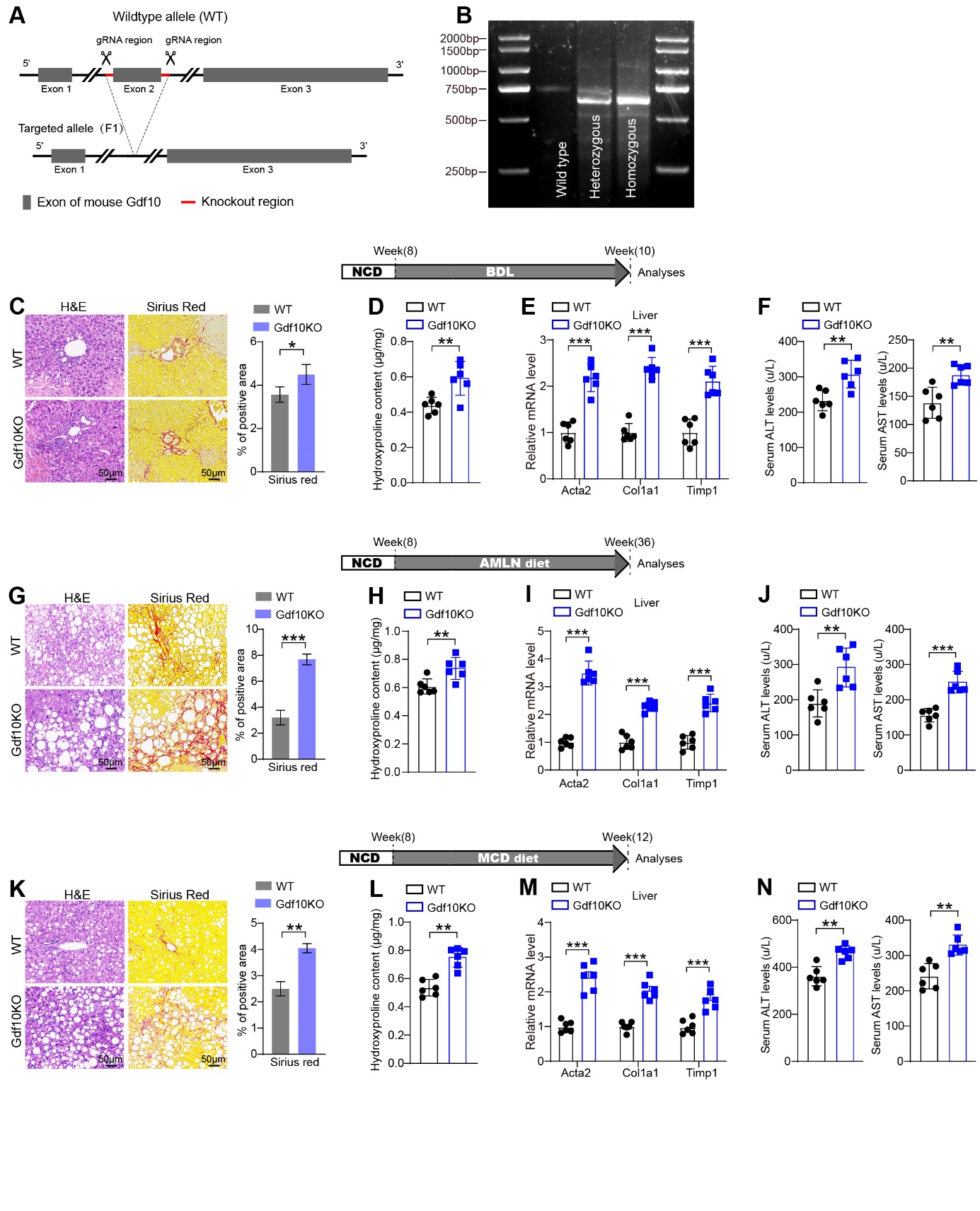
 **Figure S4.** *Gdf10* knockout accelerates liver fibrosis in mouse model. A) Generation of Gdf10KO mice. B) PCR-based genotyping of Gdf10KO mice. C-F) Representative images of H&E and Sirius Red staining of liver (C) (n = 3), total liver collagen content (D) (n = 6), indicated genes expression in liver (E) (n = 6), and serum ALT and AST levels (F) (n = 6) in WT and Gdf10KO mice; mice were subjected to the BDL for 2 weeks, scale bars, 50 µm. G-J) Representative images of H&E and Sirius Red staining of liver (G) (n = 3), total liver collagen content (H) (n = 6), indicated genes expression in liver (I) (n = 6), and serum ALT and AST levels (J) (n = 6) in WT and Gdf10KO mice; mice fed with chow or AMLN diet for 28 weeks, scale bars, 50 µm. K-N) Representative images of H&E and Sirius Red staining of liver (K) (n = 3), total liver collagen content (L) (n = 6), indicated genes expression in liver (M) (n = 6), and serum ALT and AST levels (N) (n = 6) in WT and Gdf10KO mice; mice fed with chow or MCD diet for 4 weeks, scale bars, 50 µm. Data are mean ± SEM. * P < 0.05, ** P < 0.01, *** P < 0.001 by the two-tailed Student's t-test.


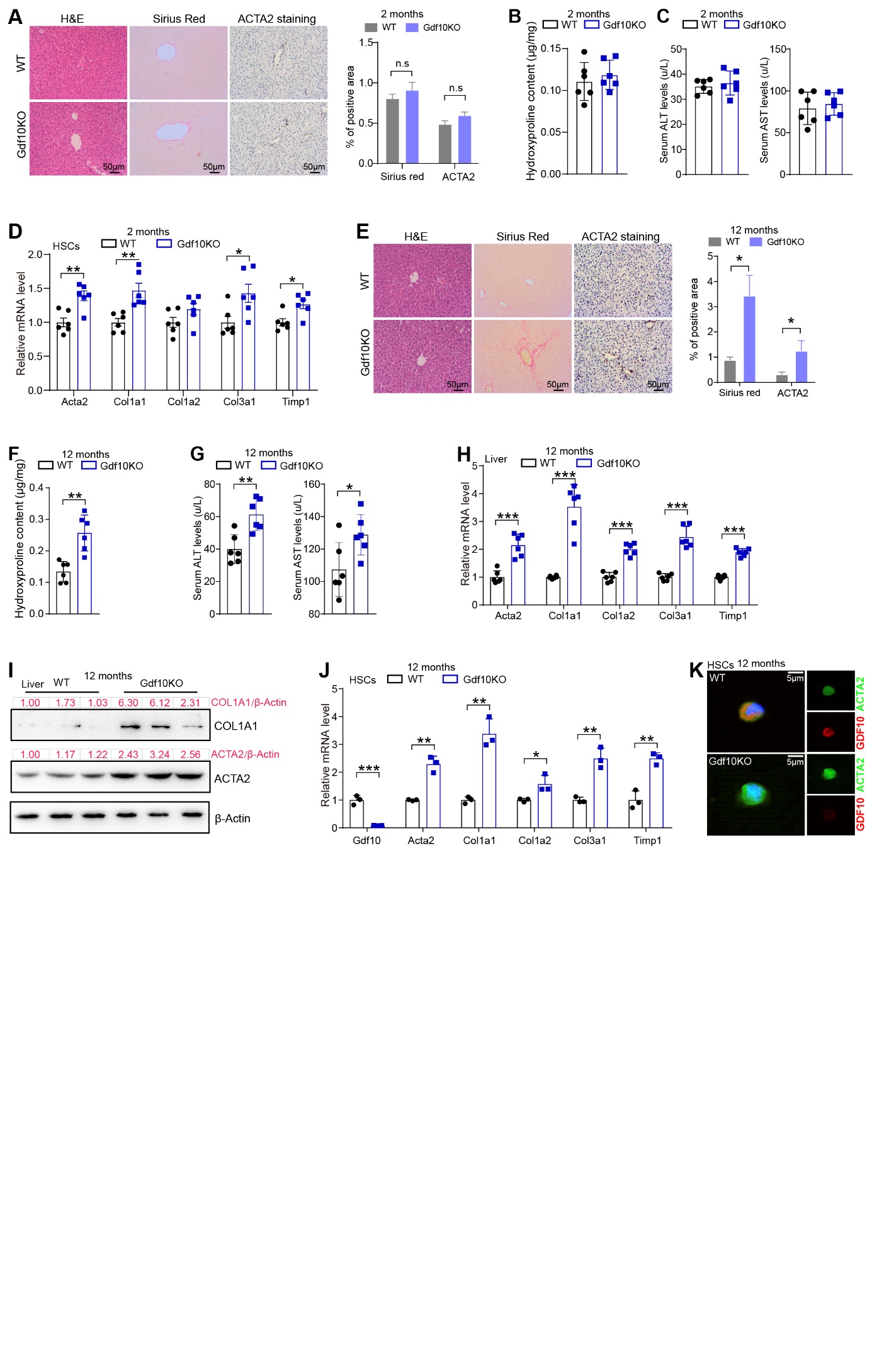
 **Figure S5.** *Gdf10* knockout results in spontaneous liver fibrosis with HSC activation. A) Representative images of H&E, Sirius Red, and ACTA2 IHC staining in the liver of 2-month-old WT and Gdf10KO mice, scale bars, 50 µm (n = 3). B, C) Measurement of the total liver collagen content (B) and serum ALT and AST levels (C) in the mice treated as in (A) (n = 6). D) qPCR analysis of indicated genes in the HSCs from mice treated as in (A) (n = 6). E) Representative images of H&E, Sirius Red, and ACTA2 IHC staining in the liver of 12-month-old WT and Gdf10KO mice, scale bars, 50 µm (n = 3). F, G) Measurement of the total liver collagen content (F) and serum ALT and AST levels (G) in the mice treated as in (E) (n = 6). H, I) qPCR (n = 6) (H) and Western blot (I) analysis of indicated genes in the HSCs from mice treated as in (E). J, K) qPCR (n = 3) (J) and IF staining (K) analysis of indicated genes in the HSCs isolated from mice treated as in (E).Data are mean ± SEM. * P < 0.05, ** P < 0.01, *** P < 0.001 by the two-tailed Student's t-test.


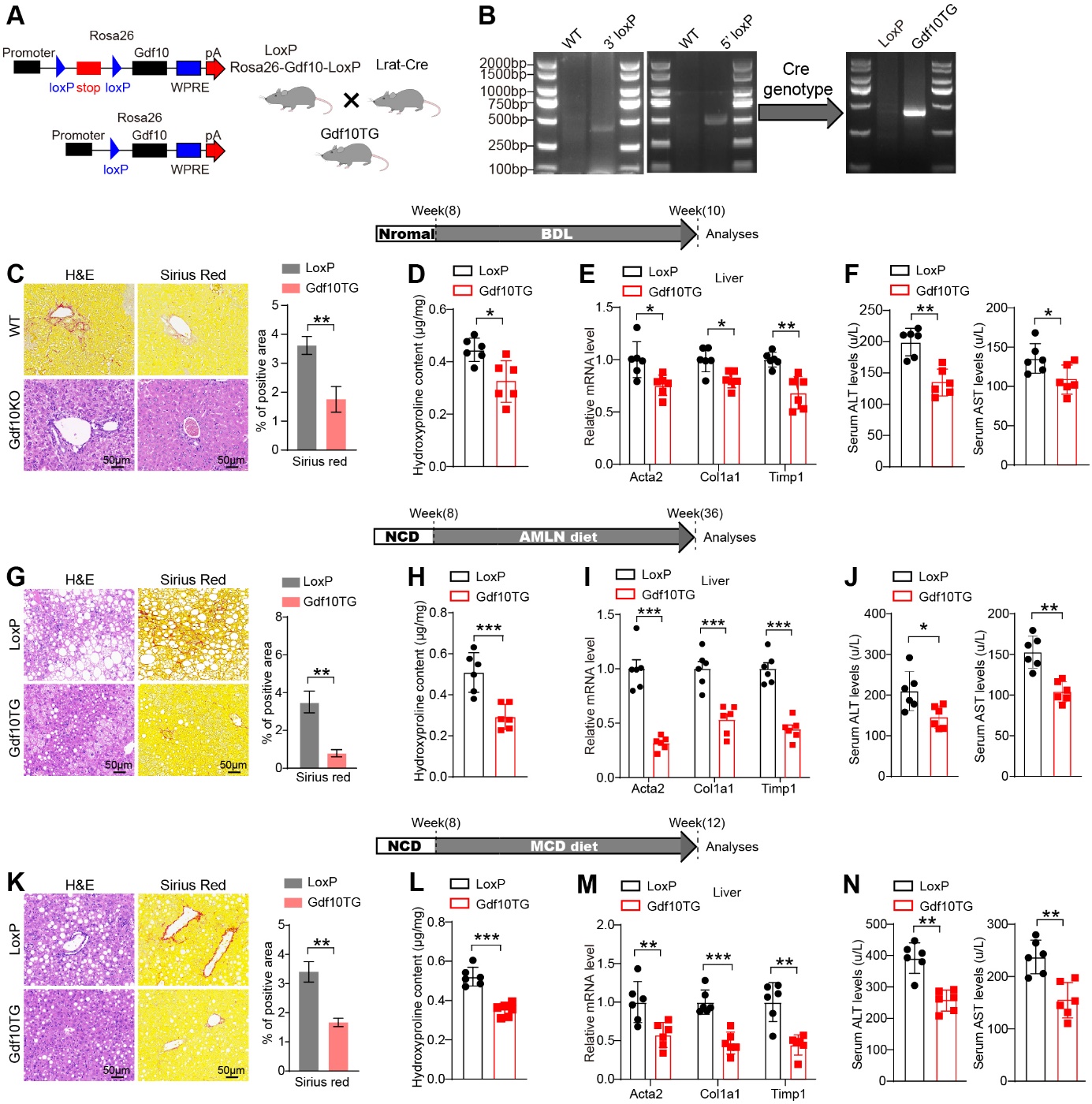


**Figure S6.** GDF10 attenuates liver fibrosis in mouse model. A) Generation of Gdf10TG mice. B) PCR-based genotyping of Gdf10TG mice. C-F) Representative images of H&E and Sirius Red staining of liver (C) (n = 3), total liver collagen content (D) (n = 6), indicated genes expression in liver (E) (n = 6), and serum ALT and AST levels (F) (n = 6) in LoxP and Gdf10TG mice; mice were subjected to the BDL for 2 weeks, scale bars, 50 µm. G-J) Representative images of H&E and Sirius Red staining of liver (G) (n = 3), total liver collagen content (H) (n = 6), indicated genes expression in liver (I) (n = 6), and serum ALT and AST levels (J) (n = 6) in LoxP and Gdf10TG mice; mice fed with chow or AMLN diet for 28 weeks, scale bars, 50 µm. K-N) Representative images of H&E and Sirius Red staining of liver (K) (n = 3), total liver collagen content (L) (n = 6), indicated genes expression in liver (M) (n = 6), and serum ALT and AST levels (N) (n = 6) in LoxP and Gdf10TG mice; mice fed with chow or MCD diet for 4 weeks, scale bars, 50 µm. Data are mean ± SEM. * P < 0.05, ** P < 0.01, *** P < 0.001 by the two-tailed Student's t-test.


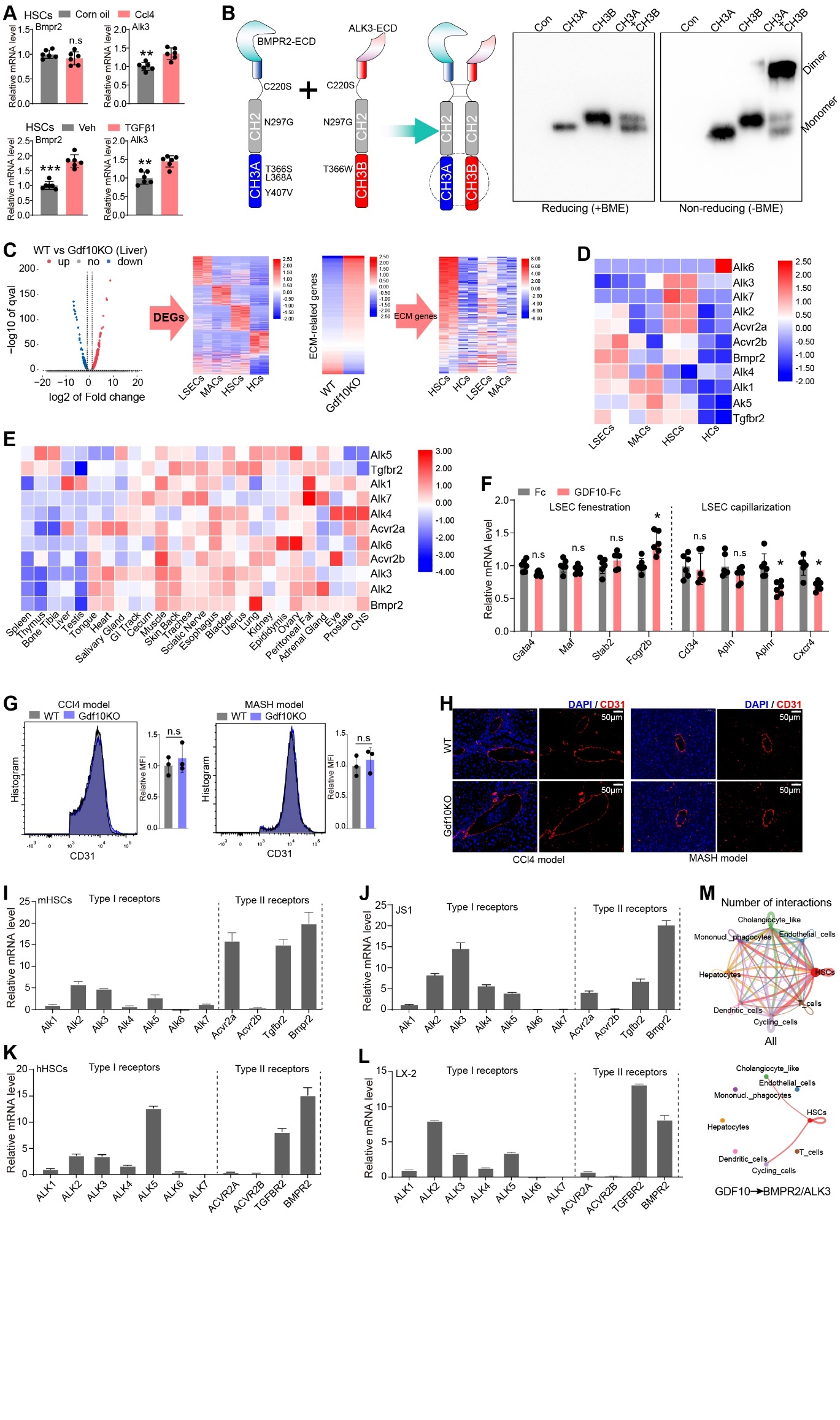
 **Figure S7.** Characterization of BMPR2:ALK3-Fc fusion protein and GDF10 exerts its antifibrotic function mainly target HSCs. A) qPCR analysis of indicated genes in the HSCs isolated from normal and CCl4-induced fibrotic liver, or treated with Veh or TGFβ1. B) Schematic overview of the BMPR2:ALK3-Fc fusion protein (left) and Western blot analysis (right) of indicated samples from under non-reducing (without β-mercaptoethanol; – BME) and reducing (+ BME) conditions. C) DEGs in WT and Gdf10KO mice liver and enrichment of these genes in LSECs, MACs, HSCs, and HCs from normal liver, data from GSE150699. D) Heat map representation of the expression of TGFβ receptors in LSECs, MACs, HSCs, and HCs from normal liver, data from GSE150699. E) Heat map representation of the expression of TGFβ receptors in different mouse tissues, data from *Gene Network.* F) qPCR analysis of indicated genes in the LSECs treated with GDF10-Fc or Fc. G) Flow cytometry analysis the number of endothelial cells in WT and Gdf10KO mice treated with CCl4 or fed with AMLN diet. H) IF staining analysis the distribution of endothelial cells in WT and Gdf10KO mice treated with CCl4 or fed with AMLN diet. I, J) qPCR analysis of TGFβ receptor superfamily in the primary mouse HSCs (I) and JS1 cells (J). K, L) Expression of all members of the TGFβ receptor superfamily in the primary human HSCs (K), data from GSE119606 and LX-2 (L), data from GSE151251. M) CellChat analysis the intercellular communication between normal liver cells, data from GSE171904. Data are mean ± SEM. * P < 0.05, ** P < 0.01, *** P < 0.001 by the two-tailed Student's t-test.


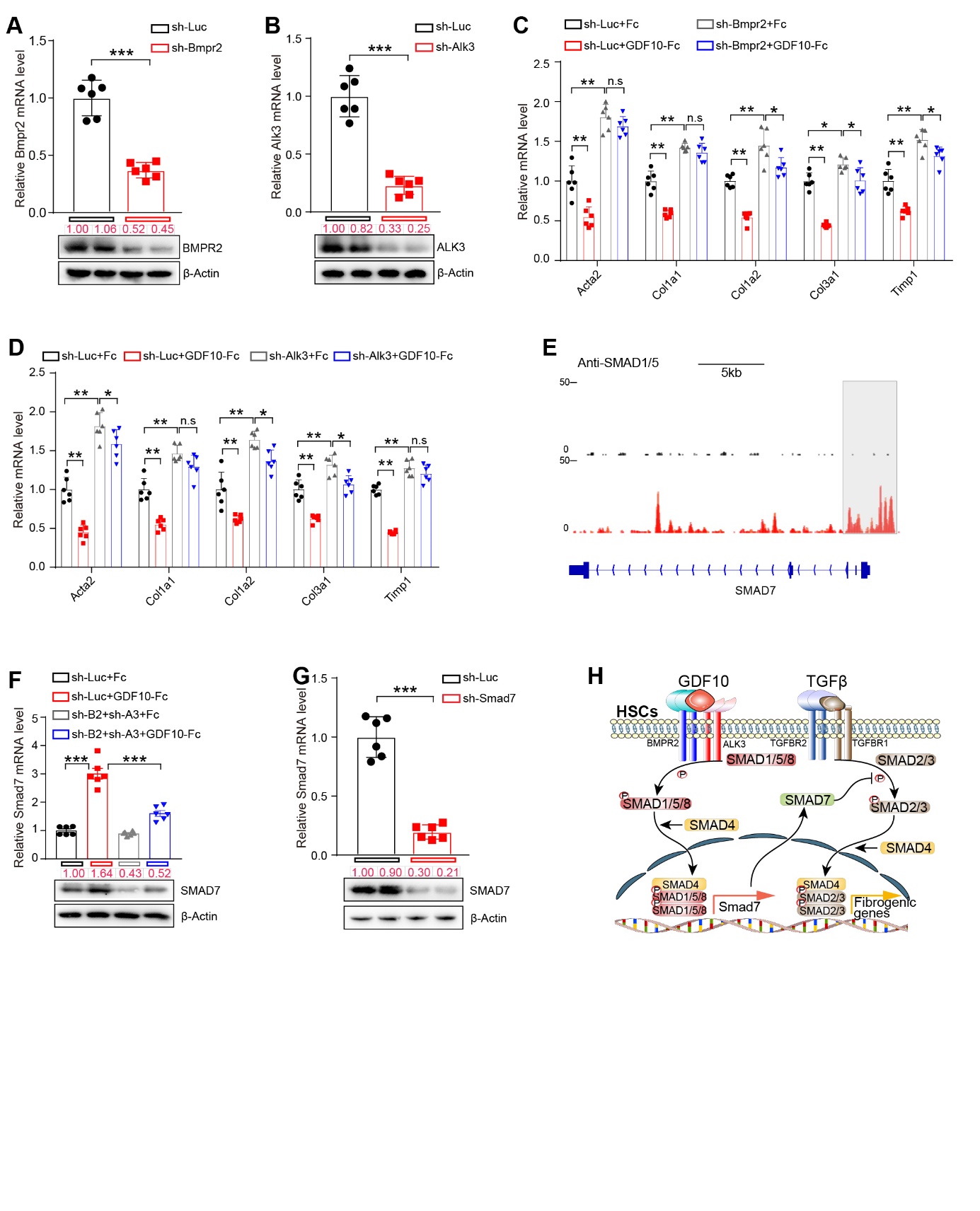
 **Figure S8.** GDF10 inhibits HSC activation via the BMPR2/ALK3-SMAD1/5/8-SMAD7 signaling pathway. A) qPCR (n = 6) (top) and Western blot (bottom) analysis of BMPR2 mRNA and protein levels in the HSCs infected with LV-sh-Luc or LV-sh-Bmpr2 for 36 h. B) qPCR (n = 6) (top) and Western blot (bottom) analysis of ALK3 mRNA and protein levels in the HSCs infected with LV-sh-Luc or LV-sh-Alk3 for 36 h. C) qPCR (n = 6) analysis of indicated genes in the primary mouse HSCs infected with LV-sh-Luc or LV-sh-Bmpr2 for 24 h and then treated with TGFβ1 plus Fc or GDF10-Fc for another 24 h. D) qPCR (n = 6) analysis of indicated genes in the primary mouse HSCs infected with LV-sh-Luc or LV-sh-Alk3 for 24 h and then treated with TGFβ1 plus Fc or GDF10-Fc for another 24 h. E) Representative genome browser screenshots of SMAD1/5 ChIP-seq at the *SMAD7* loci, genomic coordinates in hg19, data from GSE104682. F) qPCR (n = 6) (top) and Western blot (bottom) analysis of SMAD7 mRNA and protein levels in the HSCs infected with LV-sh-Luc or LV-sh-Bmpr2 and LV-sh-Alk3 for 24 h and then treated with TGFβ1 plus Fc or GDF10-Fc for another 24 h. G) qPCR (n = 6) (top) and Western blot (bottom) analysis of SMAD7 mRNA and protein levels in the HSCs infected with LV-sh-Luc or LV-sh-Smad7 for 36 h. H) The proposed model of GDF10 prevents HSC activation. Data are mean ± SEM. * P < 0.05, ** P < 0.01, *** P < 0.001 by the two-tailed Student's t-test (A, B, G), one-way ANOVA (F), or two-way ANOVA (C, D).


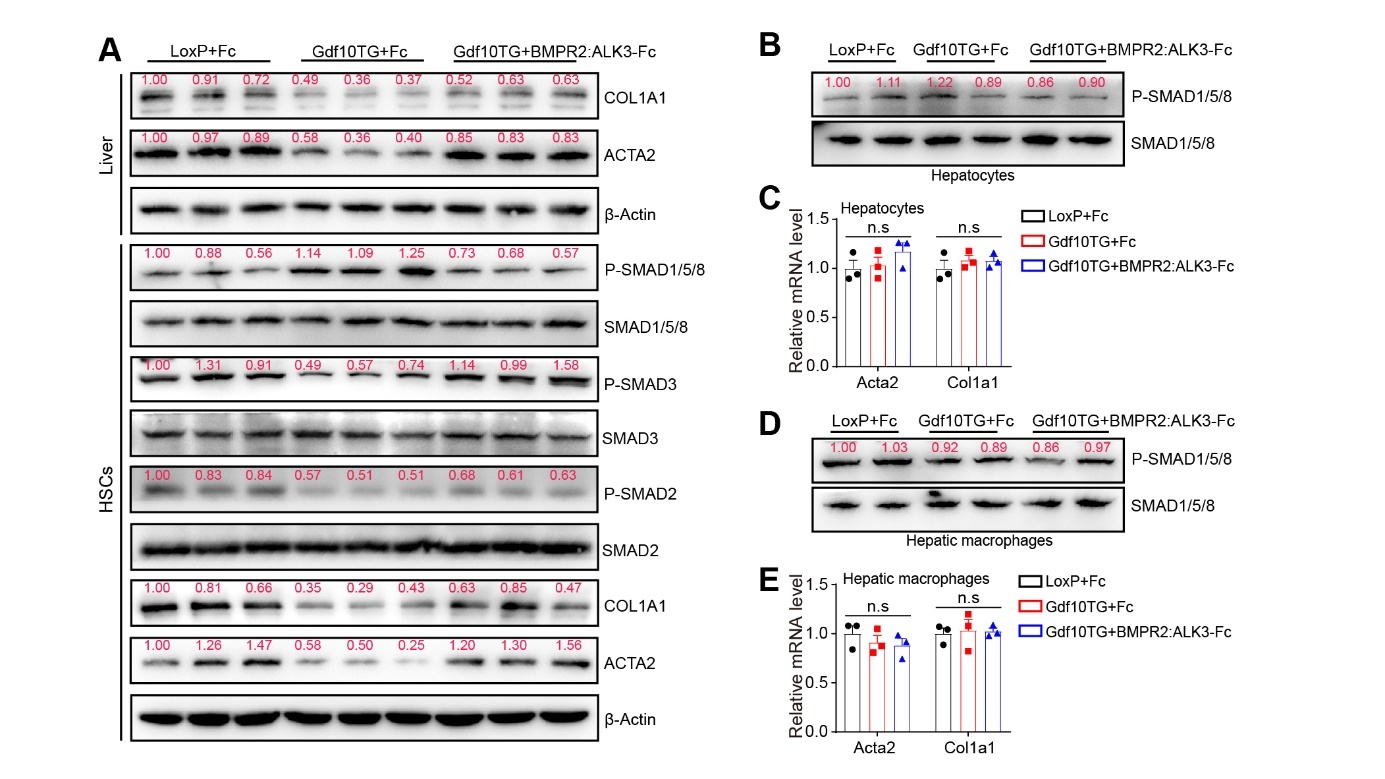
 **Figure S9.** BMPR2:ALK3-Fc prevents the antifibrotic effect of GDF10 in the liver. A) Western blot analysis of indicated protein phosphorylation in the liver and HSCs from LoxP and Gdf10TG mice treated as in (Figure 7A). B) Western blot analysis of SMAD1/5/8 phosphorylation in HCs from LoxP and Gdf10TG mice treated as in (Figure 7A). C) qPCR analysis of indicated genes in HCs from LoxP and Gdf10TG mice treated as in (Figure 7A). D) Western blot analysis of SMAD1/5/8 phosphorylation in MACs from LoxP and Gdf10TG mice treated as in (Figure 7A). E) qPCR analysis of indicated genes in MACs from LoxP and Gdf10TG mice treated as in (Figure 7A). Data are mean ± SEM. no significance (n.s) by the two-way ANOVA.


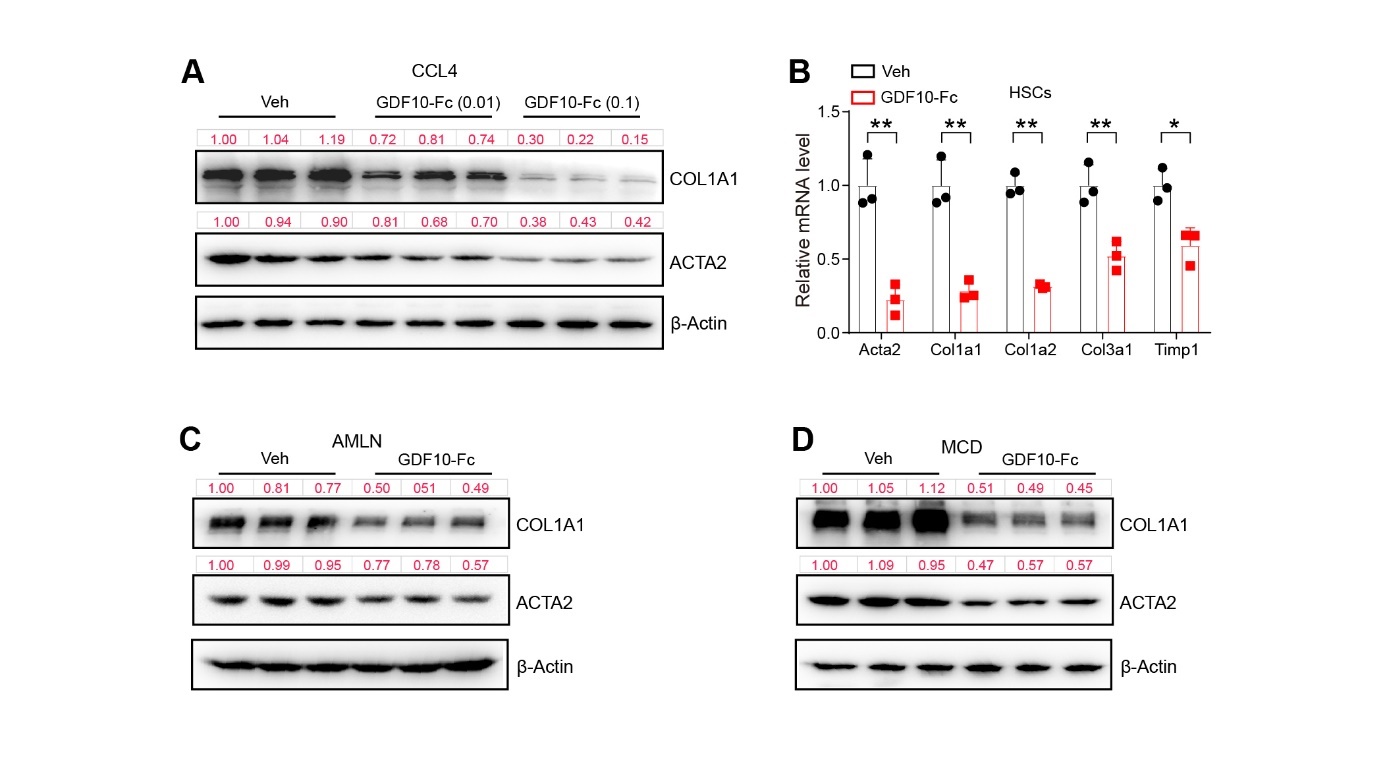
 **Figure S10.** Therapeutic administration of GDF10-Fc improves hepatic fibrosis. A) Western blot analysis of indicated genes in the livers of CCl4-induced liver fibrosis model mice with or without GDF10-Fc treatment. B) qPCR analysis of indicated genes in the HSCs from mice treated as in (A). C) Western blot analysis of indicated genes in the livers of AMLN diet-induced liver fibrosis model mice with or without GDF10-Fc treatment. D) Western blot analysis of indicated genes in the livers of MCD diet-induced liver fibrosis model mice with or without GDF10-Fc treatment. Data are mean ± SEM. * P < 0.05, ** P < 0.01 by the two-tailed Student's t-test.

**Table 1**

| Primers for quantitative PCR analysis | | |
| --- | --- | --- |
| Gene symbol | Forward primer (5’→ 3’) | Reverse primer (5’→ 3’) |
| *mActa2* | TGACCCAGATTATGTTTGAGACC | CCAGAGTCCAGCACAATACCA |
| *mCol1a1* | CATGAGCCGAAGCTAACCC | TGTGGCAGATACAGATCAAGC |
| *mCol1a2* | CAGCGGTGAAGAAGGAAAGA | ATTGCCAGGAGGACCCATTA |
| *mCol3a1* | TGCTCCTGTGCTTCCTGATG | GACCTGGTTGTCCTGGAAGG |
| *mTimp1* | CCAGAACCGCAGTGAAGAGT | GAGTACGCCAGGGAACCAAG |
| *mGdf10* | GTACAACCGAAGAGGTGCTC | GTCTTGCATGGAAGTCAAGTT |
| *mAlk1* | TGGCAGGAAATCTCACCACAT | GCAGTTCACCAGCTTGGAAGG |
| *mAlk2* | GCTAATGATGATGGCTTTCCC | CTTCACAGTGGTCCTCGTTCC |
| *mAlk3* | AGCCTGTCTGTTCATCATTTC | GGTATCCTCTGGTGCTAAAGT |
| *mAlk4* | CTGACACCATAGACATTGCTCC | GTCAAAGTGCTTCATGTTGATTG |
| *mAlk5* | TTTATGATATGACAACATCAGGGTC | CAAACTTCTCCAAACCGACCT |
| *mAlk6* | TGACTCTGGAATGCCTGTTGT | TTCTTTGATGAGGAATGGGAG |
| *mAlk7* | CGTGACCAAAACCGAATGTT | TCAGCTCTGTGGGACCAAGT |
| *mAcvr2a* | GCATTGCTGACTTTGGGTTGG | TGCGTCCCTTTGGAAGTTTAT |
| *mAcvr2b* | GCCGCTTTGGCTGCGTTTGG | TCACTCTGCCACGACTGCTTG |
| *mBmpr2* | TGACCTGGATAACCTGAAGCT | CCATCAAAGGCACTCTGTAAAT |
| *mTgfbr2* | CTGTCCACTTGCGACAACCAG | GGCAAACCGTCTCCAGAGTAATG |
| *mSmad7* | GTGTTGCTGTGAATCTTACGGG | CATTGGGTATCTGGAGTAAGGAG |
| *mHnf4a* | CTGCCTCAAAGCCATCATCTT | TAATCCTCCAGGCTCACTTGC |
| *mAdgre1* | GGATTCTGGGAAGTTTGGATAA | AGAGCAGTTGGAATACTTAGGG |
| *mCd31* | CTCACGCTGGTGCTCTATG | CGAGGTGGTGCTGATGTCC |
| *mNgfr* | ATTCCTGTCTATTGCTCCATCTTG | GCTGTTGGCTCCTTGTTTATTTT |
| *mDes* | GGAGCGTGACAACCTGATAGAC | CTAGAGTGGCTGCATCCACATC |
| *mStab2* | ACTCTAACCATCAAGACGGAG | GGACTGGATTTTAAGCGAAT |
| *mGata4* | CTGGAAGACACCCCAATCTC | GGTAGTGTCCCGTCCCATCT |
| *mFcgr2b* | TGTGGACAGCCGTGCTAAATC | TCAGTGTCACCGTGTCTTCCT |
| *mCd34* | GGGTATCTGCCTGGAACTAAG | CAGACACTAGCACCAGCATCA |
| *mApln* | GCGGACCGAGTTGCAGCATGA | TGTTCCATCTGGAGGCAACATC |
| *mAplnr* | TCGGCTAAGGCTGCGAGTCAG | GCGTCTGTGGAACGGAACACC |
| *mMaf* | CTGCAGCCCATCTGGCGGAGC | AGGGGACTGGTGGGCAGGTCG |
| *mCxcr4* | ACGGCTGTAGAGCGAGTGTTG | CAGGGTTCCTTGTTGGAGTCATA |
| *m36B4* | GAGGAATCAGATGAGGATATGGGA | AAGCAGGCTGACTTGGTTGC |
| *hACTA2* | CCGGGACTAAGACGGGAATC | TTGTCACACACCAAGGCAGT |
| *hCOL1A1* | CTGCTGGTCCTAAGGGTGAG | GCTCCAGGGCGACCTCT |
| *hCOL1A2* | TGGCTTCAAAGGCATTAGGGG | TCACCAGGGGCACCAGGTTCACCCT |
| *hCOL3A1* | TCAAAGGTGAACGTGGCAGT | GGGGTCCTGGGTTACCATTAC |
| *hTIMP1* | CTGTGAGGAATGCACAGTGTT | GGGACTGGAAGCCCTTTTCA |
| *hGDF10* | GCACAGGCTCTATGAGAAGTACA | ATGGAAGTCAGGTTGAAGAAATAC |
| *h36B4* | GTTCACCAAGGAGGACCTCAC | CTGGCACAGTGACTTCACATG |

**Table 2.**

| List of specific primers used for ChIP PCR analysis**.** | | |
| --- | --- | --- |
| Targeted Gene | Forward primer (5’→ 3’) | Reverse primer (5’→ 3’) |
| *Smad7* | ATCTTGTCCCCGAGCTGCGC | ACGCCGCCGAGCGCTCGGC |

**Table 3.**

|  | GSE130123 | | fold | P value | GSE119340 | | fold | P value | GSE156918 | | fold | P value | GSE156918 | | fold | P value |
| --- | --- | --- | --- | --- | --- | --- | --- | --- | --- | --- | --- | --- | --- | --- | --- | --- |
|  | Con | CCl4 |  |  | Con | AMLN |  |  | Con | MCD |  |  | Con | PBC |  |  |
| Tiam2 | 7.881 | 6.153 | -1.73 | 0.009 | 9.569 | 7.872 | -1.7 | 0.032 | 7.951 | 6.363 | -1.59 | 0.014 | 8.127 | 6.3 | -1.83 | 0.003 |
| Chst11 | 5.104 | 7.715 | 2.611 | 0.008 | 4.302 | 7.792 | 3.491 | 0.004 | 0.627 | 5.256 | 4.63 | 0.002 | 4.578 | 7.195 | 2.617 | 7E-04 |
| Tes | 8.25 | 9.604 | 1.354 | 0.031 | 6.553 | 8.415 | 1.861 | 0.002 | 4.021 | 7.306 | 3.285 | 1E-07 | 6.99 | 9.19 | 2.2 | 1E-04 |
| Sox4 | 9.905 | 11.42 | 1.516 | 5E-04 | 5.916 | 8.462 | 2.546 | 0.004 | 5.663 | 7.507 | 1.844 | 0.004 | 7.572 | 9.792 | 2.22 | 0.002 |
| Col1a2 | 13.51 | 16.4 | 2.891 | 0.003 | 9.503 | 13.32 | 3.813 | 4E-05 | 7.587 | 9.646 | 2.059 | 3E-04 | 9.198 | 13.14 | 3.942 | 4E-05 |
| Cand2 | 6.736 | 8.625 | 1.889 | 2E-04 | 4.438 | 6.437 | 1.999 | 0.013 | 2.923 | 5.666 | 2.743 | 5E-04 | 2.122 | 3.858 | 1.736 | 0.113 |
| Col1a1 | 12.27 | 15.73 | 3.462 | 0.006 | 8.248 | 13.43 | 5.182 | 6E-05 | 5.982 | 9.216 | 3.233 | 7E-05 | 7.097 | 12.31 | 5.216 | 3E-04 |
| Ly9 | 6.963 | 8.247 | 1.284 | 0.039 | 7.576 | 9.56 | 1.984 | 0.001 | 5.371 | 7.134 | 1.764 | 7E-04 | 7.55 | 9.264 | 1.713 | 0.003 |
| Lgals3 | 13.16 | 16.27 | 3.115 | 0.015 | 8.997 | 12.26 | 3.258 | 3E-04 | 6.588 | 10.92 | 4.335 | 1E-08 | 9.599 | 12.79 | 3.191 | 3E-04 |
| Ccdc120 | 6.754 | 8.153 | 1.399 | 0.012 | 5.365 | 8.839 | 3.474 | 0.001 | 3.004 | 7.648 | 4.644 | 4E-05 | 4.551 | 6.298 | 1.747 | 0.031 |
| Eid2 | 6.405 | 7.534 | 1.129 | 0.009 | 4.723 | 5.819 | 1.097 | 0.008 | 2.932 | 5.524 | 2.592 | 2E-04 | 6.111 | 7.171 | 1.059 | 0.016 |
| Thbs1 | 7.362 | 9.418 | 2.056 | 0.005 | 6.714 | 9.259 | 2.545 | 0.001 | 3.701 | 6.178 | 2.477 | 1E-03 | 3.158 | 6.318 | 3.16 | 3E-04 |
| S100a11 | 10.91 | 12.35 | 1.449 | 0.028 | 7.307 | 10.85 | 3.542 | 6E-04 | 4.933 | 8.93 | 3.997 | 1E-06 | 9.451 | 12.26 | 2.805 | 1E-04 |
| Thbs2 | 7.667 | 9.96 | 2.293 | 3E-04 | 7.586 | 9.939 | 2.353 | 0.002 | 4.469 | 6.224 | 1.755 | 0.003 | 7.599 | 9.629 | 2.029 | 0.005 |
| Thbs3 | 7.996 | 9.287 | 1.291 | 1E-04 | 4.831 | 6.544 | 1.713 | 0.004 | 2.672 | 5.72 | 3.048 | 4E-06 | 4.5 | 6.044 | 1.544 | 0.018 |
| Gipc2 | 6.8 | 9.734 | 2.934 | 1E-03 | 2.603 | 8.877 | 6.274 | 8E-04 | 1.539 | 7.315 | 5.777 | 1E-05 | 3.783 | 9.193 | 5.41 | 9E-05 |
| Fxyd5 | 10.37 | 11.98 | 1.614 | 0.046 | 8.212 | 10.07 | 1.855 | 3E-04 | 5.878 | 7.86 | 1.982 | 6E-06 | 9.733 | 11.45 | 1.712 | 2E-04 |
| Arl11 | 6.113 | 8.049 | 1.937 | 0.007 | 5.298 | 7.873 | 2.575 | 0.002 | 2.416 | 5.5 | 3.084 | 4E-06 | 6.653 | 9.111 | 2.458 | 5E-04 |
| Pdgfb | 9.176 | 10.63 | 1.457 | 0.004 | 6.944 | 9.004 | 2.06 | 0.006 | 3.645 | 6.152 | 2.507 | 3E-06 | 6.278 | 8.18 | 1.902 | 0.004 |
| Hhip | 5.811 | 4.422 | -1.39 | 0.002 | 9.103 | 5.544 | -3.56 | 0.009 | 5.063 | 6.367 | 1.303 | 0.001 | 4.88 | 2.73 | -2.15 | 0.006 |
| Islr | 9.436 | 11.28 | 1.849 | 0.002 | 8.121 | 9.861 | 1.741 | 0.003 | 4.969 | 6.631 | 1.661 | 3E-04 | 8.505 | 9.813 | 1.308 | 0.01 |
| Slamf9 | 8.947 | 10.55 | 1.6 | 0.022 | 6.219 | 8.826 | 2.607 | 0.001 | 3.743 | 6.283 | 2.54 | 2E-04 | 7.013 | 8.594 | 1.581 | 0.004 |
| Dbn1 | 8.142 | 9.343 | 1.2 | 4E-04 | 6.645 | 8.598 | 1.952 | 3E-04 | 4.641 | 6.045 | 1.405 | 0.002 | 7.459 | 8.912 | 1.453 | 2E-04 |
| B3gnt8 | 6.391 | 7.552 | 1.161 | 0.017 | 5.757 | 7.187 | 1.43 | 0.004 | 4.155 | 5.426 | 1.271 | 0.003 | 4.931 | 6.973 | 2.041 | 0.011 |
| Tmeff1 | 5.632 | 7.684 | 2.052 | 0.007 | 3.117 | 5.127 | 2.01 | 0.009 | -0.65 | 2.768 | 3.415 | 0.012 | -1.74 | 2.572 | 4.311 | 0.083 |
| Eln | 7.089 | 8.592 | 1.503 | 0.003 | 6.557 | 10.1 | 3.545 | 7E-04 | 4.631 | 7.388 | 2.757 | 2E-04 | 6.443 | 8.109 | 1.666 | 0.003 |
| Rhoh | 10.82 | 9.805 | -1.02 | 0.014 | 5.982 | 7.424 | 1.442 | 0.02 | 3.472 | 5.09 | 1.618 | 0.006 | 7.3 | 8.584 | 1.284 | 0.006 |
| Ctsk | 9.024 | 14.4 | 5.374 | 9E-07 | 6.012 | 8.542 | 2.53 | 0.005 | 2.708 | 5.652 | 2.945 | 5E-04 | 6.118 | 9.267 | 3.149 | 0.001 |
| Rhoc | 13.55 | 15.1 | 1.545 | 0.003 | 9.7 | 10.85 | 1.147 | 0.025 | 6.734 | 8.938 | 2.204 | 3E-05 | 10.95 | 12.45 | 1.496 | 7E-04 |
| Tinag | 7.137 | 11.44 | 4.3 | 0.002 | -1.82 | 5.732 | 7.555 | 0.036 | -0.44 | 6.32 | 6.759 | 1E-04 | -3.32 | 5.321 | 8.643 | 0.028 |
| Mrc2 | 6.624 | 8.579 | 1.955 | 0.005 | 6.84 | 9.639 | 2.799 | 0.005 | 4.29 | 5.941 | 1.651 | 1E-03 | 5.365 | 7.218 | 1.853 | 0.002 |
| Prmt2 | 9.713 | 10.81 | 1.096 | 0.019 | 5.971 | 7.309 | 1.337 | 0.002 | 3.789 | 5.742 | 1.953 | 5E-04 | 6.622 | 7.586 | 0.965 | 0.002 |
| Ltbp3 | 9.479 | 11.45 | 1.974 | 2E-05 | 7.522 | 9.625 | 2.104 | 0.001 | 4.859 | 6.77 | 1.912 | 7E-04 | 6.372 | 8.382 | 2.011 | 2E-04 |
| Ltbp2 | 9.484 | 13.02 | 3.535 | 1E-04 | 5.298 | 8.62 | 3.322 | 5E-04 | 2.902 | 6.073 | 3.171 | 3E-04 | 4.522 | 7.692 | 3.17 | 0.006 |
| Scd1 | 16.4 | 17.86 | 1.466 | 0.038 | 17.4 | 19.23 | 1.823 | 0.008 | 17.44 | 10.87 | -6.57 | 4E-04 | 15.52 | 13.87 | -1.65 | 0.014 |
| Airn | 6.112 | 7.357 | 1.245 | 6E-05 | 3.915 | 5.808 | 1.894 | 0.002 | 2.64 | 6.256 | 3.616 | 5E-05 | 5.702 | 6.785 | 1.083 | 0.003 |
| Glipr1 | 8.685 | 10.7 | 2.017 | 0.014 | 5.452 | 7.919 | 2.467 | 0.005 | 3.48 | 6.141 | 2.661 | 6E-04 | 7.677 | 10.13 | 2.452 | 2E-04 |
| C1qtnf6 | 5.434 | 6.731 | 1.297 | 0.002 | 6.575 | 8.451 | 1.876 | 0.004 | 3.558 | 6.354 | 2.796 | 2E-05 | 7.282 | 9.768 | 2.487 | 0.002 |
| Mmp12 | 6.214 | 11.74 | 5.521 | 3E-04 | 6.439 | 12.02 | 5.584 | 3E-04 | 2.322 | 11.13 | 8.809 | 2E-06 | 4.705 | 12.12 | 7.418 | 2E-05 |
| Col3a1 | 13.33 | 16.03 | 2.706 | 0.003 | 9.991 | 14.27 | 4.279 | 1E-04 | 8.576 | 10.4 | 1.824 | 0.005 | 10.66 | 14.55 | 3.891 | 2E-05 |
| Mmp13 | 8.734 | 9.978 | 1.244 | 0.016 | 3.361 | 8.676 | 5.314 | 0.002 | 0.61 | 6.511 | 5.902 | 7E-04 | 3.536 | 6.601 | 3.065 | 0.002 |
| Slc6a8 | 11.15 | 13.38 | 2.233 | 2E-04 | 8.957 | 10.43 | 1.472 | 7E-04 | 5.649 | 8.867 | 3.218 | 1E-05 | 8.479 | 10.53 | 2.053 | 0.001 |
| Obsl1 | 6.455 | 7.963 | 1.508 | 0.028 | 5.151 | 7.213 | 2.063 | 0.003 | 3.55 | 5.084 | 1.534 | 0.018 | 4.008 | 6.031 | 2.024 | 0.019 |
| Emp1 | 10.04 | 13.08 | 3.04 | 0.002 | 6.82 | 10.78 | 3.965 | 7E-04 | 3.523 | 7.644 | 4.121 | 2E-05 | 7.851 | 10.94 | 3.085 | 1E-06 |
| Kcne4 | 5.447 | 7.102 | 1.655 | 0.004 | 4.917 | 6.02 | 1.103 | 0.103 | -0.04 | 3.169 | 3.212 | 0.033 | 6.18 | 7.632 | 1.452 | 0.002 |
| Emp3 | 10.38 | 11.8 | 1.417 | 0.035 | 7.778 | 9.309 | 1.531 | 0.006 | 4.728 | 6.964 | 2.235 | 1E-04 | 8.624 | 10.45 | 1.827 | 0.001 |
| Mfge8 | 14.04 | 16.16 | 2.113 | 0.004 | 10.3 | 11.7 | 1.394 | 0.014 | 7.255 | 10.59 | 3.337 | 1E-05 | 9.444 | 12.55 | 3.103 | 2E-04 |
| Spire2 | 4.385 | 5.453 | 1.068 | 0.011 | 2.558 | 6.464 | 3.905 | 0.007 | -0.39 | 4.959 | 5.347 | 0.002 | 3.871 | 7.179 | 3.308 | 0.001 |
| Tlr13 | 11.22 | 12.54 | 1.324 | 0.036 | 7.946 | 9.92 | 1.974 | 0.003 | 4.538 | 7.304 | 2.766 | 1E-06 | 8.332 | 10.12 | 1.792 | 0.007 |
| Matn2 | 8.539 | 9.825 | 1.285 | 0.02 | 6.614 | 8.698 | 2.084 | 0.002 | 4.247 | 5.76 | 1.513 | 0.001 | 4.303 | 5.687 | 1.384 | 0.023 |
| Ahnak | 11.08 | 12.48 | 1.397 | 0.001 | 9.952 | 11.03 | 1.073 | 0.004 | 7.699 | 9.269 | 1.57 | 0.002 | 4.843 | 6.825 | 1.981 | 0.025 |
| Lat2 | 7.03 | 9.174 | 2.145 | 0.017 | 5.409 | 8.647 | 3.239 | 2E-04 | 2.779 | 5.892 | 3.113 | 7E-05 | 5.706 | 8.533 | 2.828 | 3E-04 |
| Epdr1 | 6.797 | 7.813 | 1.016 | 0.003 | 4.556 | 7.149 | 2.593 | 3E-04 | 1.804 | 6.443 | 4.639 | 2E-07 | 6.131 | 8.019 | 1.888 | 1E-04 |
| Pik3r5 | 7.504 | 9.442 | 1.938 | 0.041 | 7.189 | 8.552 | 1.363 | 0.002 | 3.789 | 6.328 | 2.539 | 2E-04 | 4.09 | 5.69 | 1.6 | 0.067 |
| Gcnt1 | 6.088 | 7.555 | 1.467 | 0.019 | 6.016 | 7.657 | 1.641 | 0.003 | 3.262 | 5.02 | 1.758 | 4E-04 | 6.461 | 7.991 | 1.53 | 0.007 |
| Cd36 | 11.15 | 12.52 | 1.371 | 0.025 | 12.72 | 14.49 | 1.769 | 0.002 | 9.076 | 13.08 | 4 | 2E-04 | 11.85 | 13.79 | 1.934 | 0.028 |
| Lynx1 | 8.248 | 9.532 | 1.284 | 0.001 | 6.475 | 8.412 | 1.937 | 4E-04 | 3.721 | 6.767 | 3.047 | 1E-04 | 7.435 | 8.647 | 1.211 | 0.003 |
| Ddr1 | 7.622 | 9.952 | 2.33 | 0.001 | 6.829 | 9.547 | 2.718 | 6E-04 | 3.998 | 7.673 | 3.675 | 8E-06 | 6.981 | 9.312 | 2.33 | 0.004 |
| Robo2 | 8.248 | 10.01 | 1.763 | 3E-04 | 7.149 | 9.275 | 2.126 | 2E-04 | 4.799 | 6.56 | 1.761 | 0.001 | 7.362 | 8.801 | 1.439 | 0.006 |
| Gprc5b | 6.619 | 9.05 | 2.431 | 0.003 | 5.663 | 8.614 | 2.951 | 2E-04 | 1.675 | 4.612 | 2.937 | 5E-04 | 2.91 | 5.441 | 2.531 | 8E-04 |
| Gpnmb | 7.865 | 14.87 | 7.004 | 2E-04 | 6.307 | 12.68 | 6.372 | 7E-05 | 1.921 | 11.2 | 9.283 | 9E-08 | -0.16 | 11.37 | 11.53 | 0.004 |
| Tmem132a | 9.479 | 10.76 | 1.283 | 0.003 | 7.602 | 9.172 | 1.57 | 0.004 | 3.443 | 4.794 | 1.351 | 0.015 | 5.255 | 6.732 | 1.477 | 1E-03 |
| Afp | 9.803 | 12.9 | 3.1 | 0.002 | 6.378 | 7.528 | 1.15 | 0.075 | 4.702 | 11.65 | 6.946 | 7E-05 | 7.345 | 8.526 | 1.18 | 0.047 |
| Ccdc3 | 5.858 | 8.069 | 2.21 | 0.002 | 7.306 | 10.07 | 2.768 | 5E-04 | 4.482 | 6.903 | 2.421 | 3E-04 | 7.639 | 8.941 | 1.301 | 0.01 |
| Ntf5 | 5.747 | 7.646 | 1.898 | 8E-05 | 0.756 | 3.786 | 3.03 | 0.008 | -1.59 | 1.178 | 2.77 | 0.015 | 0.165 | 4.729 | 4.564 | 0.086 |
| Rem1 | 8.552 | 9.922 | 1.37 | 0.004 | 4.596 | 6.081 | 1.485 | 0.008 | 2.283 | 3.469 | 1.186 | 0.021 | 3.934 | 6.315 | 2.38 | 7E-04 |
| Ifi27l2b | 12.25 | 14.5 | 2.249 | 0.023 | 8.818 | 12.07 | 3.25 | 4E-04 | 5.423 | 9.339 | 3.916 | 3E-05 | 10.7 | 12.95 | 2.243 | 3E-05 |
| Pak1 | 7.202 | 8.755 | 1.553 | 0.026 | 7.028 | 9.356 | 2.327 | 0.001 | 5.535 | 8.348 | 2.813 | 8E-05 | 8.136 | 9.657 | 1.521 | 3E-04 |
| Fkbp10 | 10.52 | 12.02 | 1.495 | 0.01 | 6.286 | 8.416 | 2.13 | 0.001 | 3.535 | 6.116 | 2.581 | 7E-04 | 5.56 | 7.149 | 1.588 | 0.003 |
| Ckb | 8.952 | 10.95 | 1.996 | 0.008 | 8.8 | 10.39 | 1.589 | 0.006 | 5.949 | 7.723 | 1.774 | 6E-04 | 9.443 | 11.32 | 1.873 | 5E-04 |
| Cd63 | 13.23 | 16.2 | 2.969 | 0.001 | 8.731 | 12.33 | 3.602 | 0.007 | 5.902 | 10.72 | 4.818 | 3E-06 | 8.718 | 12.12 | 3.405 | 4E-05 |
| Cx3cl1 | 8.043 | 10.25 | 2.21 | 0.001 | 5.999 | 9.086 | 3.086 | 0.003 | 2.418 | 5.248 | 2.83 | 0.01 | 5.814 | 8.395 | 2.581 | 0.008 |
| Fabp4 | 12.78 | 13.8 | 1.022 | 0.009 | 9.438 | 11.31 | 1.871 | 0.003 | 7.185 | 8.575 | 1.39 | 0.002 | 10.87 | 12.07 | 1.202 | 0.008 |
| Capg | 11.66 | 13.85 | 2.192 | 0.016 | 8.086 | 10.84 | 2.757 | 0.003 | 5.084 | 8.98 | 3.896 | 2E-07 | 8.689 | 11.46 | 2.771 | 1E-04 |
| Timp2 | 10.15 | 11.86 | 1.702 | 0.002 | 10.51 | 11.94 | 1.425 | 0.006 | 7.34 | 8.77 | 1.43 | 0.002 | 10.48 | 11.66 | 1.177 | 0.003 |
| Cd200r1 | 7.33 | 9.31 | 1.98 | 0.011 | 6.159 | 7.745 | 1.586 | 0.014 | 3.631 | 5.644 | 2.012 | 3E-04 | 5.384 | 6.972 | 1.588 | 4E-04 |
| Dync2li1 | 7.142 | 8.477 | 1.335 | 8E-04 | 5.173 | 7.729 | 2.555 | 9E-04 | 3.159 | 5.385 | 2.226 | 0.001 | 7.482 | 9.042 | 1.56 | 0.003 |
| Plekho1 | 11.92 | 13.15 | 1.226 | 0.04 | 8.579 | 9.842 | 1.264 | 0.006 | 6.489 | 7.895 | 1.406 | 2E-04 | 10.14 | 11.71 | 1.57 | 0.002 |
| Plp2 | 8.725 | 10.37 | 1.646 | 0.023 | 5.79 | 7.655 | 1.866 | 0.01 | 2.816 | 5.197 | 2.382 | 0.003 | 6.757 | 8.628 | 1.871 | 4E-04 |
| Tmem139 | 7.044 | 8.659 | 1.615 | 5E-04 | 2.756 | 5.531 | 2.775 | 0.008 | 1.924 | 5.681 | 3.757 | 3E-04 | 5.965 | 6.931 | 0.967 | 0.036 |
| Gdf10 | 10.02 | 11.51 | 1.487 | 3E-04 | 8.863 | 10.24 | 1.372 | 0.008 | 6.354 | 7.899 | 1.544 | 7E-04 | 7.12 | 8.388 | 1.268 | 0.013 |
| Ncf2 | 4.906 | 6.013 | 1.107 | 0.027 | 8.374 | 10.15 | 1.781 | 0.002 | 5.959 | 7.975 | 2.016 | 3E-05 | 7.73 | 9.478 | 1.748 | 0.007 |
| Tceal5 | 6.939 | 10.05 | 3.107 | 2E-04 | -3.32 | 2.433 | 5.755 | 0.005 | -2.75 | 6.151 | 8.896 | 1E-06 | -1.74 | 3.883 | 5.621 | 0.038 |
| Sh3bgrl3 | 11.05 | 12.1 | 1.053 | 0.019 | 9.299 | 10.9 | 1.602 | 0.015 | 6.746 | 8.7 | 1.954 | 3E-06 | 10.37 | 12.05 | 1.677 | 5E-05 |
| Cxcr4 | 6.533 | 8.288 | 1.755 | 0.013 | 6.982 | 8.765 | 1.783 | 0.002 | 2.819 | 5.993 | 3.174 | 4E-04 | 8.002 | 10.06 | 2.057 | 0.009 |
| Ppic | 11.21 | 12.96 | 1.746 | 0.001 | 7.6 | 9.452 | 1.852 | 0.004 | 5.27 | 6.536 | 1.266 | 0.002 | 9.438 | 11.53 | 2.093 | 8E-04 |
| Crip1 | 13.57 | 14.79 | 1.225 | 0.015 | 7.663 | 9.826 | 2.163 | 0.001 | 6.184 | 8.321 | 2.137 | 7E-04 | 10.8 | 12.34 | 1.536 | 0.016 |
| Efemp2 | 11.72 | 12.89 | 1.173 | 0.003 | 7.933 | 9.546 | 1.612 | 0.001 | 5.584 | 6.961 | 1.377 | 0.003 | 8.415 | 9.566 | 1.151 | 5E-04 |
| Clip2 | 6.436 | 7.899 | 1.463 | 0.008 | 7.516 | 9.173 | 1.657 | 3E-04 | 5.349 | 7.662 | 2.312 | 2E-05 | 7.239 | 8.525 | 1.287 | 0.005 |
| Cd83 | 9.452 | 11.53 | 2.079 | 0.028 | 7.834 | 9.304 | 1.47 | 0.042 | 5.159 | 6.963 | 1.804 | 0.002 | 9.204 | 11.19 | 1.984 | 0.024 |
| Tnxb | 10.26 | 12.12 | 1.859 | 0.004 | 9.256 | 10.69 | 1.439 | 0.023 | 6.29 | 8.13 | 1.841 | 8E-04 | 8.018 | 9.2 | 1.182 | 0.001 |
| Tubb2b | 6.149 | 7.778 | 1.629 | 0.012 | 2.412 | 6.847 | 4.435 | 0.002 | -0.77 | 4.844 | 5.611 | 7E-04 | 5.389 | 8.634 | 3.245 | 1E-04 |
| Aldh18a1 | 6.09 | 7.355 | 1.265 | 0.014 | 6.359 | 8.451 | 2.093 | 0.002 | 4.419 | 8.334 | 3.915 | 7E-05 | 7.017 | 8.541 | 1.524 | 0.003 |
| Col5a2 | 6.639 | 8.593 | 1.954 | 0.001 | 7.679 | 10.42 | 2.737 | 4E-04 | 5.303 | 7.229 | 1.926 | 0.004 | 6.874 | 10.19 | 3.318 | 1E-06 |
| Hpgds | 6.691 | 8.266 | 1.574 | 0.006 | 6.444 | 9.03 | 2.586 | 0.006 | 4.453 | 7.488 | 3.035 | 5E-06 | 7.233 | 8.664 | 1.431 | 0.003 |
| Adamts2 | 11.98 | 13.82 | 1.843 | 6E-04 | 8.214 | 10.75 | 2.531 | 2E-04 | 5.985 | 7.536 | 1.551 | 0.001 | 7.194 | 8.822 | 1.628 | 8E-04 |
| Lox | 5.303 | 6.873 | 1.57 | 0.033 | 3.971 | 7.772 | 3.801 | 8E-04 | 1.744 | 4.757 | 3.013 | 0.001 | 0.761 | 4.574 | 3.813 | 0.126 |
| Sdr9c7 | 10.57 | 9.57 | -1 | 0.027 | 11.2 | 9.993 | -1.2 | 0.014 | 9.381 | 8.181 | -1.2 | 0.008 | 12.36 | 10.98 | -1.37 | 1E-03 |
| Frzb | 7.685 | 9.577 | 1.892 | 0.005 | 7.124 | 9.233 | 2.108 | 1E-03 | 3.926 | 6.512 | 2.586 | 1E-05 | 6.462 | 9.245 | 2.783 | 6E-04 |
| Mtmr11 | 7.288 | 9.868 | 2.581 | 0.003 | 6.805 | 9.06 | 2.255 | 0.004 | 4.9 | 8.206 | 3.306 | 5E-04 | 10.23 | 11.09 | 0.865 | 0.065 |
| Anxa3 | 8.959 | 10.97 | 2.015 | 0.011 | 8.55 | 9.951 | 1.401 | 0.007 | 6.229 | 8.143 | 1.914 | 3E-05 | 8.689 | 10.25 | 1.563 | 0.005 |
| Olfml3 | 9.513 | 11.98 | 2.462 | 7E-04 | 8.163 | 10.97 | 2.804 | 0.003 | 5.773 | 7.747 | 1.974 | 2E-06 | 8.847 | 10.85 | 2.006 | 0.003 |
| Anxa2 | 13.81 | 15.42 | 1.613 | 0.022 | 9.598 | 12.9 | 3.3 | 2E-05 | 7.915 | 11.96 | 4.048 | 7E-07 | 10.86 | 13.24 | 2.376 | 2E-04 |
| Anxa1 | 9.843 | 12.72 | 2.872 | 0.001 | 8.144 | 10.16 | 2.011 | 7E-04 | 6.257 | 8.296 | 2.039 | 8E-04 | 9.42 | 11.5 | 2.081 | 2E-04 |
| Pqlc3 | 8.503 | 10.79 | 2.284 | 0.006 | 5.361 | 8.04 | 2.679 | 2E-04 | 1.081 | 5.164 | 4.082 | 8E-06 | 5.524 | 7.949 | 2.424 | 0.006 |
| Dpep2 | 4.478 | 7.056 | 2.578 | 0.002 | 1.128 | 7.237 | 6.109 | 0.11 | -1.92 | 3.164 | 5.083 | 0.001 | 3.348 | 6.858 | 3.51 | 0.004 |
| Lpl | 12.82 | 15.42 | 2.6 | 7E-04 | 11.09 | 13.53 | 2.444 | 0.006 | 8.775 | 12.71 | 3.938 | 3E-07 | 9.518 | 12.45 | 2.933 | 1E-04 |
| Svep1 | 8.472 | 10.7 | 2.231 | 1E-04 | 5.638 | 9.038 | 3.4 | 1E-04 | 2.097 | 4.579 | 2.482 | 0.004 | 4.323 | 6.852 | 2.528 | 0.01 |
| Nid1 | 11.98 | 12.98 | 1.004 | 0.009 | 10.11 | 11.74 | 1.621 | 4E-04 | 7.406 | 9.658 | 2.251 | 1E-04 | 7.533 | 8.745 | 1.211 | 0.014 |
| Tuba8 | 8.669 | 9.928 | 1.258 | 0.033 | 8.721 | 7.538 | -1.18 | 0.056 | 3.511 | 9.52 | 6.009 | 2E-09 | 7.011 | 9.19 | 2.178 | 2E-04 |
| Tgfb3 | 8.957 | 11.6 | 2.642 | 0.001 | 6.245 | 8.166 | 1.921 | 0.009 | 3.44 | 5.967 | 2.527 | 6E-05 | 4.904 | 6.365 | 1.461 | 0.004 |
| Pdgfrb | 10.66 | 12.37 | 1.713 | 1E-04 | 8.918 | 11.12 | 2.202 | 3E-04 | 6.982 | 8.187 | 1.204 | 0.005 | 6.885 | 8.111 | 1.226 | 9E-05 |
| Pdgfrl | 7.977 | 10.35 | 2.371 | 0.003 | 4.798 | 7.25 | 2.452 | 0.013 | 2.405 | 3.699 | 1.294 | 0.006 | 4.412 | 6.23 | 1.817 | 0.002 |
| Loxl2 | 10.6 | 12 | 1.405 | 0.014 | 7.576 | 9.84 | 2.265 | 4E-05 | 4.612 | 6.612 | 2 | 6E-04 | 5.66 | 7.313 | 1.654 | 0.007 |
| S100a6 | 11.79 | 13.4 | 1.615 | 0.042 | 6.582 | 9.536 | 2.954 | 0.007 | 4.417 | 7.242 | 2.826 | 0.006 | 9.724 | 11.83 | 2.108 | 0.007 |
| Loxl1 | 9.103 | 11.8 | 2.697 | 8E-05 | 6.439 | 9.917 | 3.478 | 0.001 | 3.996 | 6.142 | 2.146 | 1E-04 | 6.291 | 8.839 | 2.549 | 0.004 |
| Trpv2 | 10 | 11.27 | 1.271 | 0.032 | 7.513 | 8.541 | 1.028 | 0.004 | 4.434 | 6.605 | 2.171 | 2E-04 | 4.995 | 6.376 | 1.381 | 0.016 |
| S100a9 | 13.17 | 15.17 | 2 | 0.039 | 6.395 | 8.043 | 1.648 | 0.002 | 4.527 | 8.136 | 3.61 | 1E-04 | 11.21 | 12.83 | 1.617 | 0.069 |
| Metrnl | 10.93 | 12.31 | 1.379 | 0.003 | 7.784 | 8.949 | 1.165 | 0.038 | 5.352 | 6.862 | 1.51 | 2E-04 | 8.746 | 9.96 | 1.214 | 0.002 |
| Scube1 | 5.836 | 10.05 | 4.217 | 7E-05 | 4.645 | 9.357 | 4.712 | 0.003 | 0.121 | 5.266 | 5.145 | 7E-04 | 0.164 | 6.316 | 6.152 | 0.043 |
| Panx1 | 9.387 | 11.12 | 1.733 | 0.019 | 5.511 | 7.078 | 1.566 | 0.005 | 3.384 | 5.401 | 2.017 | 1E-04 | 5.671 | 7.377 | 1.707 | 9E-04 |
| Ccnd1 | 12.1 | 13.31 | 1.21 | 0.018 | 11.32 | 12.53 | 1.209 | 0.049 | 10.12 | 13.16 | 3.039 | 9E-04 | 10.6 | 9.538 | -1.06 | 0.031 |
| Col4a1 | 11.27 | 12.51 | 1.245 | 0.008 | 11.02 | 12.91 | 1.893 | 2E-04 | 9.02 | 11.28 | 2.264 | 1E-05 | 7.933 | 10.61 | 2.681 | 0.001 |
| Pstpip1 | 6.436 | 8.683 | 2.247 | 0.04 | 5.021 | 7.177 | 2.155 | 0.018 | 2.848 | 5.34 | 2.492 | 4E-04 | 6.572 | 8.287 | 1.716 | 0.007 |
| Fblim1 | 8.206 | 10.66 | 2.455 | 0.001 | 7.092 | 9.639 | 2.547 | 6E-04 | 4.363 | 6.758 | 2.396 | 7E-05 | 5.041 | 6.842 | 1.801 | 0.01 |
| Sybu | 6.021 | 7.925 | 1.905 | 0.015 | 3.675 | 6.763 | 3.088 | 0.016 | 3.762 | 7.423 | 3.661 | 0.023 | 7.419 | 6.257 | -1.16 | 0.068 |
| Fbn1 | 8.621 | 10.31 | 1.684 | 0.002 | 7.594 | 10.06 | 2.463 | 3E-04 | 5.059 | 6.948 | 1.889 | 0.002 | 5.465 | 7.165 | 1.7 | 0.003 |
| Ly6d | 10.31 | 14.39 | 4.08 | 0.002 | 7.687 | 12.14 | 4.453 | 0.001 | 4.413 | 11.76 | 7.351 | 2E-04 | 8.625 | 15.1 | 6.471 | 9E-05 |
| Cxcl14 | 9.624 | 12.1 | 2.478 | 0.004 | 6.954 | 9.625 | 2.671 | 0.002 | 4.966 | 6.911 | 1.945 | 3E-04 | 7.562 | 9.885 | 2.323 | 0.002 |
| Pdlim2 | 10.47 | 11.61 | 1.147 | 0.003 | 6.804 | 8.656 | 1.852 | 7E-04 | 4.131 | 5.693 | 1.562 | 0.005 | 8.979 | 10.06 | 1.078 | 0.024 |
| Cd300lb | 7.138 | 8.859 | 1.721 | 0.013 | 6.571 | 9.261 | 2.69 | 0.002 | 4.359 | 6.969 | 2.61 | 3E-05 | 7.712 | 9.462 | 1.751 | 0.004 |
| Itgax | 9.09 | 13.32 | 4.23 | 0.01 | 6.271 | 10.72 | 4.452 | 5E-04 | 2.996 | 8.104 | 5.107 | 1E-05 | 5.121 | 8.291 | 3.17 | 2E-04 |
| Trim47 | 11.94 | 13.04 | 1.094 | 0.01 | 8.352 | 9.538 | 1.186 | 0.011 | 5.479 | 7.255 | 1.776 | 1E-04 | 7.261 | 8.678 | 1.417 | 0.008 |
| Fam78b | 4.772 | 6.736 | 1.965 | 7E-04 | 3.247 | 5.125 | 1.878 | 0.017 | -1.28 | 1.892 | 3.168 | 0.021 | 0.162 | 4.011 | 3.849 | 0.086 |
| Cplx2 | 9.546 | 10.61 | 1.063 | 0.005 | 6.594 | 7.703 | 1.109 | 0.005 | 4.42 | 6.074 | 1.654 | 0.001 | 4.4 | 2.388 | -2.01 | 0.022 |
| Mmp2 | 8.337 | 11.97 | 3.635 | 1E-05 | 7.404 | 10.65 | 3.243 | 0.001 | 4.966 | 7.171 | 2.205 | 9E-05 | 7.659 | 10.5 | 2.844 | 2E-04 |
| Gpx8 | 9.527 | 11.03 | 1.5 | 5E-04 | 5.492 | 7.433 | 1.941 | 0.016 | 3.62 | 5.074 | 1.455 | 0.007 | 7.895 | 8.996 | 1.101 | 0.015 |
| Gpx7 | 7.539 | 8.789 | 1.25 | 0.036 | 6.56 | 7.916 | 1.357 | 0.002 | 4.518 | 7.538 | 3.02 | 2E-04 | 9.239 | 10.33 | 1.09 | 0.031 |
| Nrg1 | 8.899 | 11.33 | 2.435 | 0.014 | 3.701 | 6.916 | 3.215 | 0.016 | 2.378 | 8.003 | 5.625 | 1E-08 | 3.248 | 6.324 | 3.076 | 5E-04 |
| Podxl2 | 5.295 | 6.706 | 1.411 | 0.004 | 2.748 | 4.491 | 1.744 | 0.047 | 0.179 | 4.364 | 4.185 | 0.002 | 3.828 | 5.624 | 1.796 | 0.024 |
| Pcolce | 13.49 | 14.59 | 1.106 | 0.006 | 8.593 | 10.47 | 1.878 | 0.002 | 6.741 | 7.881 | 1.14 | 0.007 | 9.256 | 10.46 | 1.208 | 3E-04 |
| Vill | 7.988 | 10.75 | 2.76 | 0.003 | 5.768 | 7.838 | 2.07 | 0.015 | 4.179 | 6.026 | 1.847 | 0.002 | 6.676 | 8.799 | 2.124 | 7E-05 |
| Arhgap22 | 7.854 | 10.01 | 2.158 | 0.006 | 3.959 | 8.329 | 4.37 | 0.001 | 0.703 | 6.727 | 6.024 | 9E-04 | 4.255 | 7.48 | 3.225 | 6E-04 |
| Egfem1 | 7.275 | 5.95 | -1.33 | 0.004 | 5.543 | 3.443 | -2.1 | 0.049 | 3.466 | 4.517 | 1.051 | 0.027 | 6.334 | 3.358 | -2.98 | 4E-06 |
| Ccdc80 | 7.769 | 10.3 | 2.533 | 0.005 | 9.548 | 11.77 | 2.22 | 8E-04 | 7.565 | 9.111 | 1.546 | 0.001 | 8.328 | 10.86 | 2.532 | 5E-06 |
| Atp1a3 | 6.491 | 8.943 | 2.452 | 0.013 | 3.746 | 7.482 | 3.735 | 1E-04 | 0.615 | 5.697 | 5.082 | 0.002 | 4.176 | 7.062 | 2.886 | 0.001 |
| Tmem86a | 11.95 | 13.55 | 1.593 | 0.005 | 9.843 | 11.5 | 1.662 | 2E-04 | 7.05 | 9.589 | 2.539 | 3E-05 | 10.65 | 12.54 | 1.899 | 0.002 |
| P2ry6 | 10.32 | 11.72 | 1.402 | 0.04 | 7.223 | 9.071 | 1.848 | 0.002 | 4.67 | 6.153 | 1.482 | 1E-04 | 7.312 | 9.38 | 2.068 | 7E-04 |
| Ms4a7 | 9.867 | 13 | 3.138 | 0.019 | 6.261 | 9.827 | 3.566 | 9E-04 | 3.433 | 6.044 | 2.611 | 4E-05 | 7.361 | 10.59 | 3.23 | 0.002 |
| Calml4 | 8.279 | 10.26 | 1.982 | 0.002 | 5.546 | 7.213 | 1.668 | 0.003 | 3.753 | 7.759 | 4.005 | 4E-06 | 7.844 | 9.314 | 1.469 | 0.003 |
| Gpr137b | 6.329 | 8.863 | 2.534 | 0.002 | 6.481 | 8.158 | 1.677 | 0.005 | 3.854 | 6.944 | 3.09 | 4E-05 | 7.17 | 8.925 | 1.755 | 0.003 |
| Col6a1 | 11.22 | 13.95 | 2.725 | 2E-04 | 9.345 | 11.64 | 2.298 | 3E-04 | 6.54 | 8.457 | 1.917 | 3E-04 | 8.759 | 10.63 | 1.874 | 5E-05 |
| Uap1l1 | 10.72 | 12.12 | 1.393 | 0.001 | 9.308 | 11.75 | 2.439 | 9E-05 | 5.802 | 9.981 | 4.18 | 2E-07 | 8.486 | 11.55 | 3.062 | 4E-05 |
| Col6a2 | 8.218 | 10.7 | 2.484 | 1E-04 | 8.249 | 11.2 | 2.953 | 3E-04 | 5.895 | 7.621 | 1.726 | 0.003 | 6.776 | 8.346 | 1.57 | 0.005 |
| Lum | 12.59 | 15.28 | 2.694 | 5E-04 | 9.503 | 12 | 2.495 | 0.003 | 7.179 | 9.016 | 1.837 | 6E-05 | 10.09 | 11.49 | 1.4 | 0.005 |
| Mfap2 | 8.404 | 11.02 | 2.613 | 2E-04 | 3.383 | 6.417 | 3.034 | 0.001 | -0.46 | 3.617 | 4.073 | 0.006 | 3.346 | 7.28 | 3.935 | 0.001 |
| Slc13a2 | 9.946 | 8.839 | -1.11 | 0.035 | 6.251 | 4.146 | -2.11 | 1E-03 | 5.48 | 2.871 | -2.61 | 0.001 | 6.125 | 3.229 | -2.9 | 0.003 |
| Plek | 11.04 | 13.04 | 2.001 | 0.045 | 8.739 | 10.6 | 1.858 | 0.005 | 5.672 | 8.308 | 2.637 | 2E-05 | 6.659 | 8.056 | 1.397 | 0.021 |
| Tagln2 | 9.518 | 11.29 | 1.773 | 0.015 | 9.845 | 12.12 | 2.27 | 9E-04 | 7.496 | 10.17 | 2.678 | 5E-05 | 11.35 | 13.25 | 1.9 | 7E-04 |
| Rgs10 | 11.26 | 12.37 | 1.103 | 0.036 | 7.06 | 9.1 | 2.039 | 0.005 | 4.944 | 6.308 | 1.364 | 0.001 | 8.983 | 10.64 | 1.659 | 0.006 |
| Emilin1 | 8.031 | 9.392 | 1.361 | 0.002 | 10.6 | 11.76 | 1.162 | 0.006 | 8.158 | 9.473 | 1.316 | 2E-04 | 9.647 | 10.93 | 1.286 | 1E-03 |
| Ncam1 | 7.753 | 11.16 | 3.403 | 7E-04 | 3.36 | 7.192 | 3.833 | 0.002 | 1.294 | 4.467 | 3.172 | 0.021 | -1.74 | 2.899 | 4.642 | 0.023 |
| Sparcl1 | 12.23 | 15.53 | 3.306 | 3E-05 | 9.285 | 11.12 | 1.839 | 0.013 | 5.777 | 7.834 | 2.057 | 0.011 | 8.376 | 9.816 | 1.44 | 0.017 |
| Atp6v0d2 | 7.249 | 11.73 | 4.485 | 6E-04 | 7.824 | 9.693 | 1.87 | 0.004 | 6.341 | 9.72 | 3.379 | 0.005 | 8.686 | 10.29 | 1.606 | 0.09 |
| Phlda3 | 10.7 | 13.46 | 2.766 | 0.006 | 6.644 | 8.852 | 2.208 | 0.002 | 3.754 | 6.671 | 2.917 | 3E-05 | 7.164 | 10.38 | 3.221 | 3E-04 |
| Phldb1 | 10.71 | 11.77 | 1.059 | 0.003 | 8.076 | 9.665 | 1.589 | 9E-04 | 5.747 | 7.024 | 1.277 | 0.004 | 6.288 | 7.368 | 1.08 | 0.014 |
| Sh2d1b1 | 5.51 | 6.787 | 1.277 | 0.025 | 2.269 | 6.397 | 4.128 | 6E-04 | -2.17 | 3.624 | 5.792 | 4E-04 | 4.64 | 8.333 | 3.693 | 0.003 |
| Unc5b | 10.03 | 11.63 | 1.598 | 7E-04 | 9.107 | 11.19 | 2.081 | 0.004 | 5.197 | 7.702 | 2.505 | 3E-06 | 8.183 | 9.689 | 1.506 | 0.003 |
| Tnfaip8 | 8.482 | 9.741 | 1.258 | 0.012 | 7.814 | 9.208 | 1.394 | 0.009 | 5.714 | 7.102 | 1.387 | 4E-04 | 9.012 | 10.09 | 1.077 | 0.011 |
| Vim | 12.99 | 15.21 | 2.22 | 0.021 | 11.09 | 12.95 | 1.864 | 0.002 | 8.066 | 10.3 | 2.235 | 1E-04 | 11.18 | 13.2 | 2.011 | 0.001 |
| Cpt1c | 5.644 | 6.95 | 1.306 | 0.006 | 4.463 | 6.331 | 1.868 | 0.009 | 1.889 | 3.804 | 1.915 | 0.003 | 5.36 | 6.33 | 0.97 | 0.001 |
